# Revalidation and expanded description of *Mustela aistoodonnivalis* (Mustelidae: Carnivora) based on a multigene phylogeny and morphology

**DOI:** 10.1002/ece3.9944

**Published:** 2023-04-18

**Authors:** Yingxun Liu, Yingting Pu, Shunde Chen, Xuming Wang, Robert W. Murphy, Xin Wang, Rui Liao, Keyi Tang, Bisong Yue, Shaoying Liu

**Affiliations:** ^1^ College of Life Sciences Sichuan University Chengdu Sichuan China; ^2^ College of Life Sciences Sichuan Normal University Chengdu Sichuan China; ^3^ Sichuan Academy of Forestry Chengdu Sichuan China; ^4^ Reptilia Sanctuary and Education Centre Concord Ontario Canada; ^5^ Ecological Restoration and Conservation for Forest and Wetland Key Laboratory of Sichuan Province Sichuan Academy of Forestry Chengdu Sichuan China

**Keywords:** geometric morphometric, *Mustela aistoodonnivalis*, *Mustela erminea*, *Mustela nivalis*, phylogeny, second lower molar

## Abstract

The lacked‐teeth pygmy weasel, *Mustela aistoodonnivalis* Wu & Kao, 1991, was originally described as being from Taibai Mountain and Zhashui county, Shaanxi, China. Subsequently, it was considered a subspecies or synonym of *Mustela nivalis*. In a faunal survey of northwestern Sichuan, eight specimens of *M. aistoodonnivalis* were collected. A molecular phylogenetic analysis of one mitochondrial and six nuclear genes clustered the specimens as a distinct clade and not with *M. nivalis*. Morphologically, the lack of the second lower molar differentiated them from *M. nivalis*, and genetic distances were typical of discrete species. These analyses confirmed that *M. aistoodonnivalis* is an independent species in the genus *Mustela*.

## Graphical abstract

The taxonomic status of *Mustela aistoodonnivalis* is of great importance to the study of ecosystem. In this study, we determined the taxonomic status of *M. aistoodonnivalis* as an independent species through morphological and molecular biological studies and gave a detailed morphological description of *M. aistoodonnivalis*.

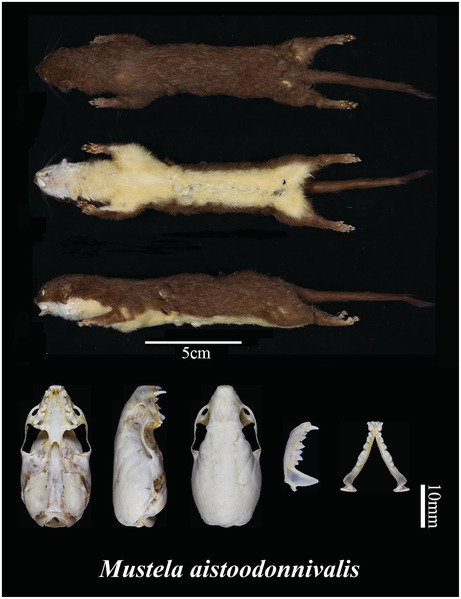

## 1 INTRODUCTION

*Mustela* is one of the most specious genera in the family Mustelidae (Carnivora). The genus occurs in Europe, North Africa, Asia, North America, and northern parts of South America. These small or middle‐sized weasels occupy diverse habitats ranging from tropical rainforests to tundra and from steppe and desert to riparian biotopes and coastal waters (Kurose et al., 2008). In Eurasia and America, sympatric congeners show substantial intraspecific geographic variation with respect to morphological, karyological, and ecological features, and sometimes these geographic forms are classified as different species (King, 1989). Mustelidae arose approximately 16.1 MYA within the Mid‐Miocene Climatic Optimum and extensively diversified in the Miocene, mostly in Asia (Sato et al., 2012). The genus *Mustela* differentiated in a stepwise fashion that is attributable in part to the geographic isolation on North America and Eurasia, as well as among peripheral insular domains, such as Taiwan and the Japanese Islands. In addition, the Eurasian continent itself was shown to have been involved in the species diversification (Hosoda et al., 2000). The genus *Mustela* exhibits much variation in terms of morphology, especially in body size.

The number of subgenera and species present in *Mustela* is a topic under discussion. The number of species ranges from 8 to 19 (Table 1). Ellerman and Morrison‐Scott (1951) recognized two subgenera and eight species and Walker et al. (1975) four subgenera and 15 species (no specific list). Youngman (1982) proposed five subgenera and 15 species and Honacki et al. (1982) 16 species. Corbet and Hill (1992) considered *Mustela* to have 17 species. Heptner and Sludskii (1992) listed two subgenera and 13 species. Wozencraft (1993) agreed with Honacki et al. (1982). Abramov (2000) listed nine subgenera and 17 species and assigned *Mustela vison* to a new genus, *Neovison*. This interpretation was followed by Wozencraft (2005), Wilson and Mittermeier (2009), and Smith and Xie (2009). Groves (2007) elevated *M. russelliana* and *M. tonkinensis* as valid species. Koepfli et al. (2017) listed 17 species in *Mustela*. Colella et al. (2021) elevated *M. haidarum* and *M. richardsonii* from *M. erminea* as valid species. Patterson et al. (2021) erected genus *Neogale* for *N. frenata*, *N. felipei*, and *N. africana*, with *Neovison vison*. San (2022) listed 19 species in the genus *Mustela*.

**TABLE 1:** Major classification systems of the genus *Mustela.*

Subgenus*	Species	EM		WA	Y		HO		HS		W1	AB		W2	WM	K	P	S
*Cabreragale*	*Mustela altaica*		*√*			*√*		*√*		*√*	*√*	*	*√*	*√*	*√*	*√*	*√*	*√*
*Cryptomustela*	*M. erminea*		***√***			*√*		*√*		*√*	*√*	*	*√*	*√*	*√*	*√*	*√*	*√*
*Gale*	*M. eversmanii*					*√*		*√*		*√*	*√*	*	*√*	*√*	*√*	*√*	*√*	*√*
*Grammogale*	*M. itatsi*			*	*	*	*			*√*		*	*√*	*√*	*√*	*√*	*√*	*√*
*Kolonokus*	*M. kathiah*		***√***					*√*		*√*	*√*	*	*√*	*√*	*√*	*√*	*√*	*√*
*Lutreola*	*M. lutreola*		***√***	*	*	*√*	*	*√*		*√*	*√*	*	*√*	*√*	*√*	*√*	*√*	*√*
*Mustela*	*M. lutreolina*	*		*	*	*√*	*	*√*	*	*√*	*√*	*	*√*	*√*	*√*	*√*	*√*	*√*
*Pocockictis*	*M. nigripes*	*				*√*	*	*√*		*√*	*√*	*	*√*	*√*	*√*	*√*	*√*	*√*
*Putorius*	*M. nivalis*	*	***√***	*	*	*√*	*	*√*	*	*√*	*√*	*	*√*	*√*	*√*	*√*	*√*	*√*
(*Vison*)	*M. nudipes*				*	*√*		*√*		*√*	*√*		*√*	*√*	*√*	*√*	*√*	*√*
	*M. putorius*		***√***			*√*		*√*		*√*	*√*		*√*	*√*	*√*	*√*	*√*	*√*
	*M. sibirica*		***√***			*√*		*√*		*√*	*√*		*√*	*√*	*√*	*√*	*√*	*√*
	*M. strigidorsa*		***√***			*√*		*√*		*√*	*√*		*√*	*√*	*√*	*√*	*√*	*√*
	*M. subpalmata*									*√*			*√*	*√*	*√*	*√*	*√*	*√*
	*M. russelliana*																	*√*
	*M. tonkinensis*																	*√*
	*Neogela africana*					*√*		*√*		*√*			*√*	*√*	*√*	*√*		*√*
	*N. felipei*					*√*		*√*		*√*	*√*		*√*	*√*	*√*	*√*		*√*
	*N. frenata*					*√*		*√*		*√*	*√*		*√*	*√*	*√*	*√*		*√*
	*N. vison*					*√*		*√*		*√*	*√*							

*Note*: EM: Ellerman and Morrison‐Scott (1951); WA: Walker et al. (1975); Y: Youngman (1982); HO: Honacki et al. (1982); HS: Heptner and Sludskii (1992); W1: Wozencraft (1993); AB: Abramov (2000); W2: Wozencraft (2005); WM: Wilson and Mittermeier (2009); K: Koepfli et al. (2017); P: Patterson et al. (2021); S: San (2022).

*Means recorded subgenus, √ means recorded species.

For China, Allen (1938) listed six species in *Mustela*: *M. altaica*, *M. erminea*, *M. eversmanii*, *M. nivalis*, *M. russelliana*, and *M. sibirica*, but Ellerman and Morrison‐Scott (1951) recognized only *M. nivalis*, *M. altaica*, *M. kathiah*, *M. sibirica*, and *M. putorius* by assigning *M. russelliana* to a subspecies of *M. nivalis*. Honacki et al. (1982) listed six species, but the species differed from those recognized by Allen (1938), including *M. altaica*, *M. erminea*, *M. eversmanii*, *M. nivalis*, *M. sibirica*, and *M. strigidorsa*. Gao (1987) confirmed eight species, adding *M. kathiah* and *M. amurensis* to the list of Honacki et al. (1982). Wu & Gao collected four specimens of *Mustela* from Taibai Mountain and Niubei Liang of Zhashui, Qingling Mountain, Shaanxi in 1991. These specimens resemble *M. nivalis*, but their tail length exceeds 1/3 of the head–body length. In these specimens, summer hairs are dark brown dorsally, and ventral hairs are yellowish and irregularly stained with rusty red patches. Second molars on the lower jaw are missing, leaving 32 teeth in total. Wu and Kao (1991) subsequently described as the new species *M. aistoodonnivalis*. Wang and Hu (1999), Wang (2003), Sheng (2005), and Pan et al. (2007) recognized *M. aistoodonnivalis*, but Wozencraft (1993), Zhang (1997), and Wilson and Mittermeier (2009) did not mention the species. Wozencraft (2005) listed *M. aistoodonnivalis* as a subspecies of *M. nivalis*. Groves (2007) considered *M. russelliana* and *M. aistoodonnivalis* as sister species, differing only in size. Smith and Xie (2009) and Lin and Motokawa (2010) agreed with this opinion. Jiang et al. (2015) listed *M. aistoodonnivalis* as a doubtful species. Wei et al. (2021) added *M. aistoodonnivalis* as a valid species. San (2022) considered *M. aistoodonnivalis* to be equivalent to *M. russelliana* and probably more closely related to *M. kathiah* than *M. nivalis*. Thus, the status of *M. aistoodonnivalis* remains highly controversial.

Herein, we report on molecular phylogenetic analyses and morphological comparisons that clarify the taxonomic status of *M. aistoodonnivalis*.

## 2 MATERIALS AND METHODS

### 2.1 Ethics statement

All samples were obtained following the Guidelines of the American Society of Mammalogists and the laws and regulations of China for the implementation of the protection of terrestrial wild animals (Sikes et al., 2011; State Council Decree, 1992). Collecting protocols and research project were approved by the Ethics Committee of the Sichuan Academy of Forestry (no specific permit number).

### 2.2 Sampling and DNA sequencing

**FIGURE 1:**
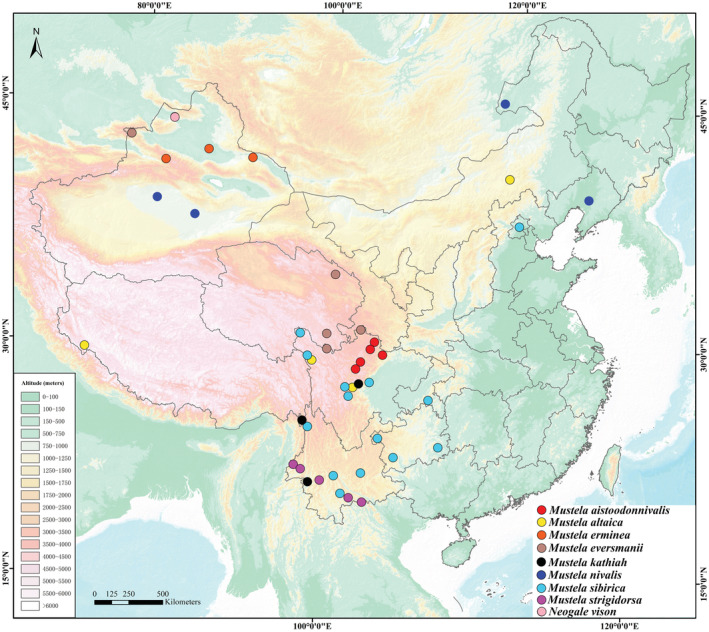
Map showing localities of specimens in the study.

For the molecular analysis, eight specimens of *M. aistoodonnivalis* were collected in faunal surveys in Heishui (Sandagu Natural Reserve), Baoxing (Fengtongzhai Natural Reserve), Li County (Miyaluo Natural Reserve), Jiuzhai (Jiuzhaigou Natural Reserve), and Pingwu (Xuebaoding Natural Reserve, Wanglang Natural Reserve), Sichuan, from 2001 to 2019. Another 16 specimens of the same family (Mustelidae) were collected from 10 localities in China, including 4 individuals of *M. altaica*, 6 individuals of *M. sibirica*, 4 individuals of *M. nivalis*, and 2 individuals of *Neogale vison* (Table 2; Figure 1). Muscle and liver tissue of all specimens were preserved in 95% ethanol, which were subsequently stored at −75°C for until DNA sequencing extraction. All specimens were identified based on external characteristics according to Pan et al. (2007) and Smith and Xie (2009).

**TABLE 2:** Samples and sequences of Mustelidae used for molecular analyses.

Species	No.	Locality	Longitude	Latitude	Altitude (m)	Genbank accession No.						
						*APOB*	*ATP7A*	*BDNF*	*CYTB*	*RAG1*	*RAG2*	*TMEM20*
*Mustela aistoodonnivalis*	SAF03190	Sichuan, Jiuzhai	102.652	30.872	3480	MT888695	MT888710	MT888725	MT888740	MT888758	MT888773	MT888788
*M. aistoodonnivalis*	SAF09530	Sichuan, Baoxing	104.04048	33.22483	3480	MT888696	MT888711	MT888726	MT888741	MT888759	MT888774	MT888789
*M. aistoodonnivalis*	SAF12703	Sichuan, Li County	102.8628	31.37425	2700	MT888706	MT888721	MT888736	MT888751	MT888769	MT888784	MT888799
*M. aistoodonnivalis*	SAF181732	Sichuan, Pingwu	104.080685	32.976603	2600	MT888707	MT888722	MT888737	MT888752	MT888770	MT888785	MT888800
*M. aistoodonnivalis*	SAF16175	Sichuan, Heishui	102.84043	32.28167	3220	MT888699	MT888714	MT888729	MT888744	MT888762	MT888777	MT888792
*M. aistoodonnivalis*	SAF191261	Sichuan, Pingwu	104.155965	32.909671	2470	ON730966	ON730973	ON730980	ON730959	ON730987	ON730994	ON731001
*M. aistoodonnivalis*	SAF191315	Sichuan, Pingwu	104.155965	32.909671	2470	ON730967	ON730974	ON730981	ON730960	ON730988	ON730995	ON731002
*M. aistoodonnivalis*	SAF191387	Sichuan, Jiuzhai	104.155965	32.909671	2470	ON730968	ON730975	ON730982	ON730961	ON730989	ON730996	——
*M. altaica*	SAF16391	Tibet, Pulan	81.16707	30.25312	3890	MT888698	MT888713	MT888728	MT888743	MT888761	MT888776	MT888791
*M. altaica*	SAF17005	Sichuan, kangding	102.00629	29.91389	3950	MT888708	MT888723	MT888738	MT888754	MT888771	MT888786	MT888801
*M. altaica*	SAF071101	Sichuan, Dege	99.22	31.877	3940	ON730970	ON730977	ON730984	ON730963	ON730991	ON730998	ON730998
*M. altaica*	SAF12193	Nei Mongol, Xilinhot	116.73953	43.62379	1180	MT888697	MT888712	MT888727	MT888742	MT888760	MT888775	MT888790
*M. nivalis*	SAF17440	Xinjiang, Xinyuan	84.030854	43.309386	1341	ON730965	ON730972	ON730979	ON730958	ON730986	ON730993	ON731000
*M. nivalis*	SAF190197	Nei Mongol, Xin Barag Youqi	117.53278	48.3746	578	ON730969	ON730976	ON730983	ON730962	ON730990	ON730997	ON731003
*M. nivalis*	SCNU01149	Liaoning, Fushun	125.043193	41.836676	564.46	MT888694	MT888709	MT888724	MT888739	MT888757	MT888772	MT888787
*M. nivalis*	SAF17306	Xinjiang, Hejing	86.41376	42.28471	1037	MT888705	MT888720	MT888735	MT888750	MT888768	MT888783	MT888798
*M. sibirica*	SAF18694	Sichuan, Hanyuan	102.878728	29.409092	2700	MT888700	MT888715	MT888730	MT888745	MT888763	MT888778	MT888793
*M. sibirica*	SAF18695	Sichuan, Hanyuan	102.878728	29.409092	2700	MT888701	MT888716	MT888731	MT888746	MT888764	MT888779	MT888794
*M. sibirica*	SAF18696	Sichuan, Hanyuan	102.878728	29.409092	2700	MT888702	MT888717	MT888732	MT888747	MT888765	MT888780	MT888795
*M. sibirica*	SAF18697	Sichuan, Hanyuan	102.878728	29.409092	2700	MT888703	MT888718	MT888733	MT888748	MT888766	MT888781	MT888796
*M. sibirica*	SAF18698	Sichuan, Hanyuan	102.878728	29.409092	2700	MT888704	MT888719	MT888734	MT888749	MT888767	MT888782	MT888797
*M. sibirica*	SAF18699	Sichuan, Hanyuan	102.878728	29.409092	2700	——	——	——	MT888753	——	——	——
*Neogela vison*	SAF182041	Xinjiang, Emin	84.184039	46.841754	847	——	——	——	MT888755	——	——	——
*N. vison*	SAF182062	Xinjiang, Emin	84.009578	46.672401	669	——	——	——	MT888756	——	——	——

*Note*: *M*. means *Mustela*, *N*. means *Neogale*.

Employing the methods of Koepfli et al. (2008), Sato et al. (2012), Law et al. (2018), and Hassanin et al. (2021), we amplified a mitochondrial gene (cytochrome b (*CYTB*) and six nuclear gene fragments (apolipoprotein B (*APOB*)), transporting alpha polypeptide (*ATP7A*), brain‐derived neurotrophic factor (*BDNF*), recombination‐activating gene 1 (*RAG1*), recombination‐activating gene 2 (*RAG2*), and transmembrane protein 20 (*TMEM20*)). During the Mammal Tree of Life Project (2011), these genes were found to provide sound phylogenetic information.

All the gene fragments were amplified with published primers (Table 3; Amrine‐Madsen et al., 2003; Irwin et al., 1991; Lindblad‐Toh et al., 2005; Murphy et al., 2000; Murphy et al., 2001). PCR amplifications were carried out in a reaction volume mixture of 25 μL containing 12.5 μL 2× Taq Master Mix (Vazyme), 1 μL each primer, 1 μL genomic DNA, and 9.5 μL double‐distilled water. PCR conditions for *CYTB* amplifications consisted of an initial denaturing step at 94°C for 5 min followed by 38 cycles of denaturation at 94°C for 45 s, annealing at 49°C for 45 s, extension at 72°C for 90 s, and then a final extension step at 72°C for 12 min. PCR conditions for the nuclear genes were basically the same as those of the *CYTB*, with a few modifications. We adjusted the annealing temperature for each nuclear gene: *APOB*: 49°C; *RAG2*, *ATP7A*, and *TMEM20*: 52°C; *RAG1*: 54°C; *BDNF*: 56°C. We checked the PCR products on a 1.0% agarose gel and subsequently purified them using ethanol precipitation. Purified PCR products were directly sequenced using the BigDye Terminator Cycle Kit v 3.1 (Applied Biosystems) and an ABI 310 Analyzer (Applied Biosystems).

**TABLE 3:** Gene symbol, primer sequences, and the best model of evolution for each gene segments used in the study.

Gene symbol	Primers sequences (5′ → 3′)	The best model	References
*CYTB*	F: CGAAGCTTGATATGAAAAACCATCGTTG	GTR+I+G	Irwin et al. (1991)
	R: GGAATTCATCTCTCCGGTTTACAAGAC		
*APOB*	F: GTGCCAGGTTCAATCAGTATAAGT	GTR+I+G	Amrine‐Madsen et al. (2003)
	R: CCAGCAAAATTTTCTTTTACTTCAA		
*ATP7A*	F: TCCCTGGACAATCAAGAAGC	HKY+G	Murphy et al. (2001)
	R: AAGGTAGCATCAAATCCCATGT		
*BDNF*	F: CATCCTTTTCCTTACTATGGTT	K80	Murphy et al. (2001)
	R: TTCCAGTGCCTTTTGTCTATG		
*RAG1*	F: GCTTTGATGGACATGGAAGAAGACAT	SYM+I+G	Teeling et al. (2000)
	R: GAGCCATCCCTCTCAATAATTTCAGG		
*RAG2*	F: TCATGGAGGGAAAACACCAAA	K80+I+G	Murphy et al. (2001)
	R: TGCACTGGAGACAGAGATTC		
*TMEM20*	F: TGGGTTTATAGGCCCCAAAG	HKY	Lindblad‐Toh et al. (2005)
	R: CACGTKGGCACATYRTTA		

F means forward primer; R means reverse primer.

To test the phylogenetic relationship among species of Chinese *Mustela* and to evaluate the species status of *M. aistoodonnivalis*, we downloaded homologous sequences of additional species of *Mustela* from GenBank for comparison (Table 4; Abramov et al., 2013; Emami‐Khoyi et al., 2016; Harding & Smith, 2009; Hosoda et al., 2000; Hosoda et al., 2011; Huang et al., 2014; Koepfli et al., 2007; Koepfli & Wayne, 1998; Kurose et al., 2008; Martinkova et al., 2007; Naoko et al., 2000; Sato et al., 2004; Sato et al., 2009; Sato et al., 2016; Sun et al., 2014; Yu et al., 2011).

**TABLE 4:** GenBank accession numbers of download sequence from NCBI.

Species	*CYTB*	*RAG2*	*ATP7A*	*BDNF*	*TMEM20*	*APOB*	*RAG1*
*Mustela altaica*	AB026100	—	—	—	—	—	—
*M. altaica*	KC815122	—	—	—	—	—	—
*M. altaica*	AB051239	—	—	—	—	—	—
*M. erminea*	K603875	—	—	—	—	—	—
*M. erminea*	K603876	—	—	—	—	—	—
*M. erminea*	K603892	—	—	—	—	—	—
*M. erminea*	K603896	—	—	—	—	—	—
*M. erminea*	K603904	—	—	—	—	—	—
*M. eversmanii*	EF987741	EF987992	EF987576	EF987620	EF988034	EF987523	EF987973
*M. eversmanii*	KT224449	—	—	—	—	—	—
*M. haidarum*	MK603010	—	—	—	—	—	—
*M. haidarum*	MK603011	—	—	—	—	—	—
*M. haidarum*	MK603012	—	—	—	—	—	—
*M. haidarum*	MK603013	—	—	—	—	—	—
*M. haidarum*	MK603014	—	—	—	—	—	—
*M. itatsi*	AB026104	—	LC124879	LC124912	—	AB285338	AB285384
*M. kathiah*	HM106320	—	—	—	—	AB285339	AB285385
*M. kathiah*	AB285331	—	—	—	—	—	—
*M. kathiah*	JQ965760	—	—	—	—	—	—
*M. lutreola*	EF987742	EF987993	EF987578	EF987622	EF988035	EF987524	EF987974
*M. lutreola*	AB026105	—	—	—	—	—	—
*M. nigripes*	EF987743	EF987994	EF987579	EF987623	EF988036	EF987525	EF987975
*M. nigripes*	GQ153574	—	—	—	—	—	—
*M. nigripes*	GQ153575	—	—	—	—	—	—
*M. nivalis*	EF987744	EF987995	EF987580	EF987624	EF988037	EF987526	EF987976
*M. nivalis*	HM106319	—	—	—	—	—	—
*M. nivalis*	AB046612	—	—	—	—	—	—
*M. nivalis*	AB564129	—	—	—	—	—	—
*M. nudipes*	EF987745	EF987996	EF987581	EF987625	—	EF987527	EF987977
*M. nudipes*	AB285332	—	—	—	—	—	—
*M. putorius*	EF987746	EF987997	EF987582	EF987626	EF988038	EF987528	EF987978
*M. putorius*	HM106318	—	—	—	—	—	—
*M. putorius*	KT693383	—	—	—	—	—	—
*M. richardsonii*	AF057127	EF987991	EF987575	EF987619	EF988033	EF987522	AB109347
*M. richardsonii*	MK603911	—	—	—	—	—	—
*M. richardsonii*	MK603912	—	—	—	—	—	—
*M. richardsonii*	MK603913	—	—	—	—	—	—
*M. richardsonii*	MK603914	—	—	—	—	—	—
*M. richardsonii*	MK603915	—	—	—	—	—	—
*M. sibirica*	EF987747	EF987998	EF987583	EF987627	EF988039	EF987529	EF987979
*M. strigidorsa*	EF987748	EF987999	EF987584	EF987628	—	EF987530	EF987980
*M. strigidorsa*	AB119078	—	—	—	—	—	—
*M. strigidorsa*	AB305635	—	—	—	—	—	—
*Neogela vison*	AF057129	DQ660281	EF987585	DQ660205	EF472443	DQ660191	DQ660268
*N. vison*	KM488625	—	—	—	—	—	—

### 2.3 Sequence analyses

All *CYTB* sequences were aligned and examined, and screening for heterozygote nuclear gene fragments was performed in Mega 5 (Tamura et al., 2011). Following Gadagkar et al. (2005), Koepfli et al. (2008), Heled et al. (2009), and Sato et al. (2016), we concatenated the six nuclear genes for analysis. Using all sequence data, phylogenetic analyses were conducted on (1) the *CYTB* data, (2) the concatenated six nuclear genes, and (3) each nuclear gene. Modeltest v3.7 (Tamura et al., 2011) was used to select the best fitting model of evolution, based on the Akaike Information Criterion in Table 3. MrBayes v3.1.2 (Ronquist & Huelsenbeck, 2003) was used for the Bayesian analysis. *Neogale vison* was selected as the outgroup. Each run was carried out with four Monte Carlo Markov chains (MCMCs) and 10,000,000 generations for single gene datasets and 30,000,000 generations for concatenated gene datasets. All runs were sampled every 10,000 generations. Convergences of runs were accepted when the average standard deviation of split frequencies fell below 0.01. Tree nodes were considered to be strongly supported when Ultrafast bootstrap values (UFBoot) were ≥95 and posterior probabilities (pp) were ≥0.95 (Heled et al., 2009).

### 2.4 Species tree and genetic distance

The *BEAST model was invoked in BEAST v1.7.5 (Drummond et al., 2012; Huelsenbeck & Rannala, 2004) using individual data analysis with complete genes in the *CYTB* + nDNA dataset to construct species trees. Model settings were selected with reference to the optimal replacement model of each gene (Table 3). For each run, we selected the birth–death speciation model. The BEAST program was set up to run 30,000,000 generations, with statistical sampling every 5000 generations.

The Kimura‐2‐parameter (K2p) interspecific distances for *CYTB* were calculated in Mega 5 (Tamura et al., 2011).

### 2.5 Linear analyses

To avoid age‐related morphological differences, we only used adult individuals for morphological analysis. No sexual dimorphism was found in Chinese *M. nivalis* and *M. aistoodonnivalis* or skull measurements because measurements of the male individual fell among those of females. Thus, all genders of *M. nivalis* and *M. aistoodonnivalis* were used for morphometric analyses. For the other species, we used adult females only for a principal component analysis (PCA). Moreover, since some species distribute in untraversed areas (*M. altaica*, *M. erminea*, and *M. nivalis*), some have the small population size or narrow distribution area in China (*M. kathiah*, *M. strigidorsa*), and some have large individual size and uneasy to be captured (*M. eversmanii*), resulting in the small sample sizes for these species. Nonetheless, 59 complete skulls of intact specimens were collected for morphological measurements, including 4 specimens of *M.altaica*, 6 specimens of *M. aistoodonnivalis*, 5 specimens of *M. erminea*, 6 specimens of *M. eversmanii*, 7 specimens of *M. kathiah*, 5 specimens of *M. nivalis*, 21 specimens of *M. sibirica*, and 5 specimens of *M. strigidorsa*. Details of collected localities and housed museums are listed in Table S1.

**FIGURE 2:**
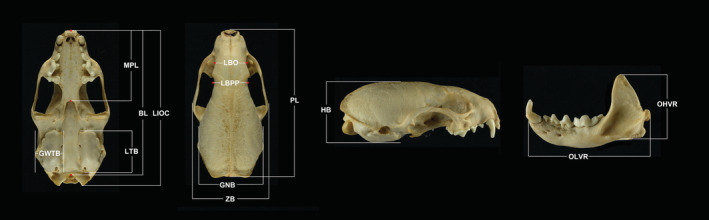
Indicators for skull linear analysis used herein. Photographs of *Mustela sibirica* (voucher No. SAF18695). For abbreviations, see Section 2 (Materials and Methods).

Following Yang et al. (2005), we measured the skulls of these specimens with a digital Vernier caliper (accuracy 0.01 mm). Thirteen measurements were taken: profile length (PL), basal length (BL), greatest neurocranium breadth (GNB), height of the braincase (HB), length between incisor and occipital condyles (LIOC), median palatal length (MPL), least breadth between the orbits (LBO), least breadth behand the postorbital process (LBPP), zygomatic breadth (ZB), length of tympanic bulla (LTB), greatest width of the tympanic bulla (GWTB), oral length of the vertical ramus (OLVR), and oral height of the vertical ramus (OHVR; Figure 2). To evaluate the morphological variation between specimens, 13 measurements were used for the principal component analysis (PCA) in SPSS 22.0 (SPSS Inc.). Before analysis, the Kaiser–Meyer–Olkin (KMO) test and Bartlett's sphere test were carried out. The KMO test was used to check the correlation and partial correlation between variables. Bartlett's test determined whether the correlation matrix was the unit matrix and the independent analysis method of each variable was invalid.

### 2.6 Geometric morphometric analyses

For the geometric morphometric analyses, dorsal, ventral, and lateral views of the cranium, as well as lateral views of the mandible, were photographed (Nikon D800 camera with a Nikon AF‐S 105 mm f 2.8G IF‐ED macro lens; Figure S1). To reduce errors, all photographs were taken by the same person. To keep the relative positions of all the objects and the camera consistent, reference points were selected on all surfaces (Yang et al., 2005).

**FIGURE 3:**
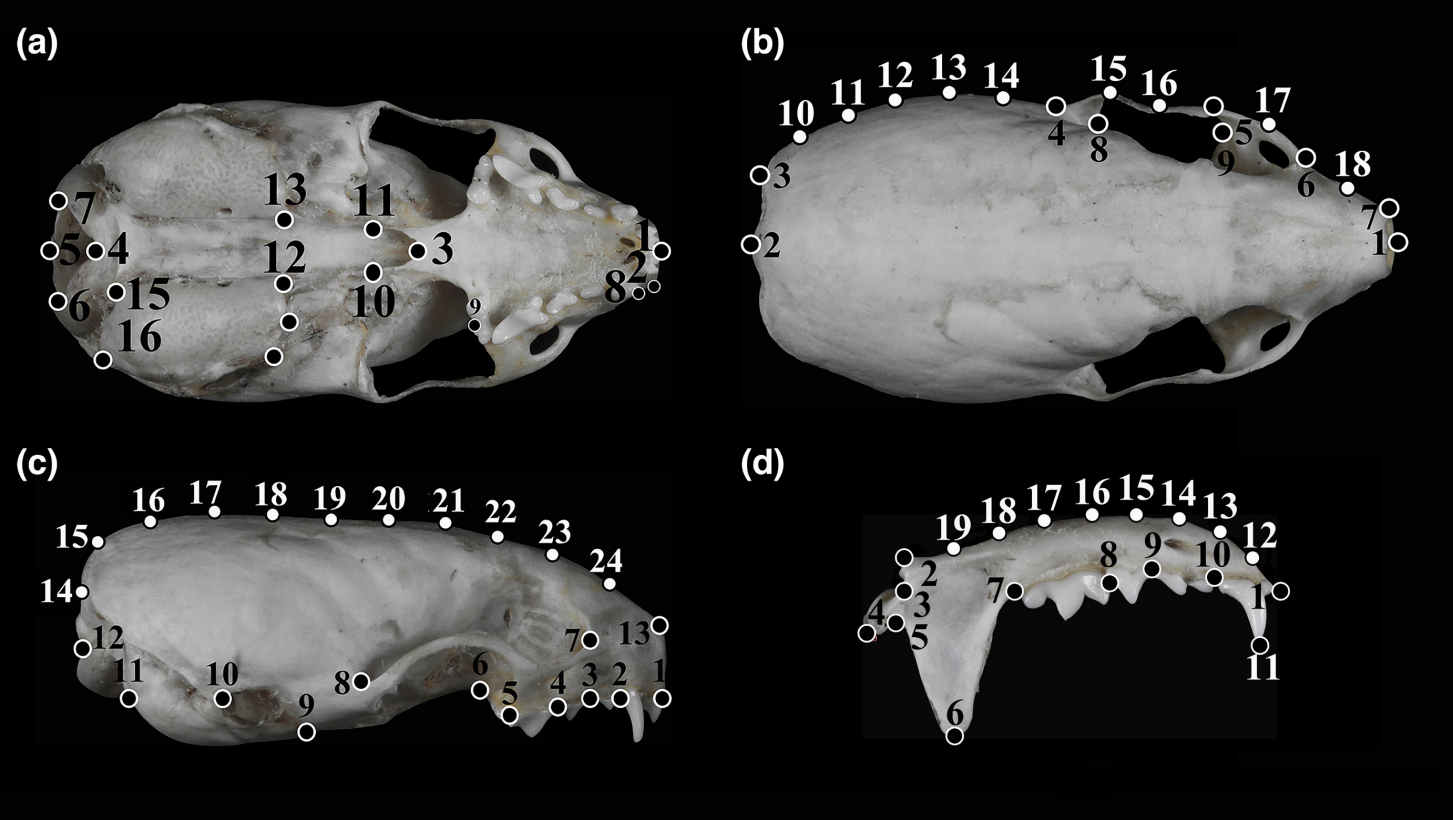
Landmarks (black circle) and semi‐landmarks (white circle) configuration sets for the dorsal (a), ventral (b), and lateral (c) views of the cranium, and lateral (d) view of the mandible. Number on the point denotes the marked order. Photographs of *Mustela aistoodonnivalis* (voucher No. SAF181732).

TPS files were produced using Tpsutil v1.65 (Zelditch et al., 2004). All photographs, which were evaluated in random order by one investigator, were scaled, identified, digitally landmarked, and semi‐landmarked using Tpsdig v2.22 (Zelditch et al., 2004). All landmarks were in accordance with the relevant animal skull marking methods and combined with the actual healing characteristics of the skulls (Rohlf, 2005). Homologous structures were chosen as landmarks. They were identified consistently in all photographs. Semi‐landmarks were useful for depicting the shape of curved lines where landmarks could not be detected (Yang et al., 2005). And semi‐landmark were equal‐diversion points based on the “Resample curve” function in tpsDig v2.22 software after drawing the objective curve along the outer edge from two landmarks (Tang et al., 2018). In the dorsal view of the cranium, semi‐landmarks 10 (SL10^#^) ‐SL14^#^ were six equal‐diversion points from landmarks 3 (L3^#^) to L4^#^, SL15^#^‐SL16^#^ were three equal‐diversion points on the outer edge of the zygomatic arch from L4^#^ to L5^#^, SL17^#^ was determined on L5^#^ to L6^#^, and SL18^#^ was determined on L6^#^ to L7^#^ (Figure 3b). In the lateral view of the cranium, semi‐landmarks SL14^#^‐SL24^#^ were 12 equal‐diversion points on the top edge curve of the cranium from L12^#^ to L13^#^ (Figure 3c). In the upper molars, SL12^#^‐ SL19^#^ were nine equal‐diversion points on the basal edge curve of the mandible from L1^#^ to L2^#^ (Figure 3d). The locations of the landmarks and semi‐landmarks are shown in Figure 3.

All configuration sets for our 59 specimens were superimposed using the least squares‐based superimposition algorithm provided by the generalized procrustes analysis (GPA) in the software Coordgen 8 (Cardini & Higgins, 2004). This procedure standardized the configuration sets for overall position, scale, and orientation, yielding a set of shape coordinates for each photograph. Subsequently, a principal component analysis (PCA) was employed, and the ordination of the aligned specimens was visualized in scatterplots using Pcagen8 (Bookstein, 1997). The shape parameters of the specimens were converted into thin lath coefficients, that is, partial warp scores. We also calculated the covariance matrix of the local warp index (Bookstein, 1997). The PC axes corresponded to eigenvectors of the variance–covariance matrix for the shape data. Eigenvalues were assumed to be proportional to the variance explained by the PCS (Yang et al., 2005). Shape deformations along the first and second principal component axes were illustrated in grids and vectors. Transformed grids represented the actual differences in the location of landmarks (or semi‐landmarks), and the length and direction of the black line on each landmark (or semi‐landmark) represented the degree and orientation of the deformation, respectively.

## 3 RESULTS

### 3.1 Sequence information

We obtained 24 new *CYTB* sequences (1140 bp), 21 *RAG1* (1064 bp), 21 *RAG2* (456 bp), 21 *ATP7A* (636 bp), 21 *BDNF* (536 bp), 21 *TMEM20* (596 bp), and 21 *APOB* (907 bp). In addition, 45 *CYTB*, 12 *RAG1*, 10 *RAG2*, 11 *ATP7A*, 11 *BDNF*, 8 *TMEM20*, and 12 *APOB* sequences were downloaded from GenBank for phylogenetic analysis. All newly obtained sequences were deposited in GenBank (Tables 2 and 4).

### 3.2 Phylogenetic analysis

The tree structure of each nuclear gene differed, and recovered nodes had very low support; these trees did not effectively explain the relationships of all species of *Mustela* (Figures S2 and S3). Bayesian reconstructions using *CYTB* and the concatenated nuclear genes displayed similar topologies and most nodes had medium or high support. Thus, the interspecific relationships of *Mustela* were explained reasonably.

**FIGURE 4:**
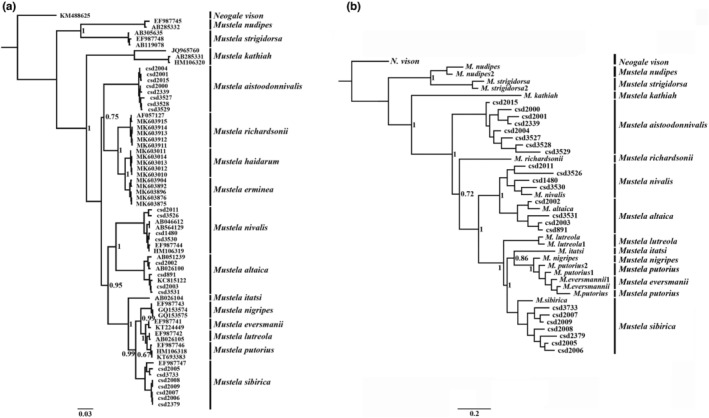
Bayesian phylogenetic analyses based on *CYTB* (a) and six nuclear genes concatenated (b). The distal of the phylogenetic tree is the sample experiment number. Numbers at nodes refer to Bayesian posterior probabilities. Scale bars represent substitutions per site.

The *CYTB* tree revealed 15 clades, corresponding to the following species: *M. nudipes, M. strigidorsa, M. kathiah, M. aistoodonnivalis, M. richardsonii, M. haidarum, M. erminea, M. altaica, M. nivalis, M. itatsi, M. sibirica, M. nigripes, M. eversmanii, M. lutreola*, and *M. putorius* (Figure 4a). Both *M. nudipus* and *M. strigidorsa* split first from the other species and clustered together with strong support (pp = 1.00). *Mustela kathiah*, which belongs to the same subgenus *Gale* as *M. altaica* and *M. nivalis*, formed an isolated clade with strong support (pp = 1.00). *Mustela aistoodonnivalis* clustered with *M. richardsonii*, *M. haidarum*, and *M. erminea*, but with very low support (pp = 0.75). Importantly, *M. aistoodonnivalis* did not cluster with *M. nivalis* as anticipated based on traditional relationships. *Mustela altaica* and *M. nivalis* were sister groups that were strongly supported (pp = 1.00). *Mustela itatsi*, *M. sibirica*, *M. nigripes*, and *M. eversmanii* also constituted a monophyletic lineage that was strongly supported (pp = 0.99), but *M. lutreola* clustered with *M. putorius*, however, and was not well supported (pp = 0.67).

The Bayesian reconstruction using the six nuclear genes concatenated (dataset 2) revealed 14 monophyletic clades (Figure 4b). In contrast to the Bayesian reconstruction using *CYTB* (dataset 1), the topological structure was slightly different, and both the analyses strongly clustered *M. aistoodonnivalis* away from *M. nivalis* (pp = 1.00). *Mustela lutreola* was monophyletic with strong support (pp = 1.00) as was *M. sibirica* (pp = 1.00). *Mustela itatsi*, *M. nigripes*, *M. eversmanii*, and *M. putorius* had mixed associations with weak support, and the samples of *M. putorius* divided into two different branches. Due to the lack of nuclear gene sequences of *M. haidarum*, its position remained uncertain.

### 3.3 Species tree and genetic distance

**FIGURE 5:**
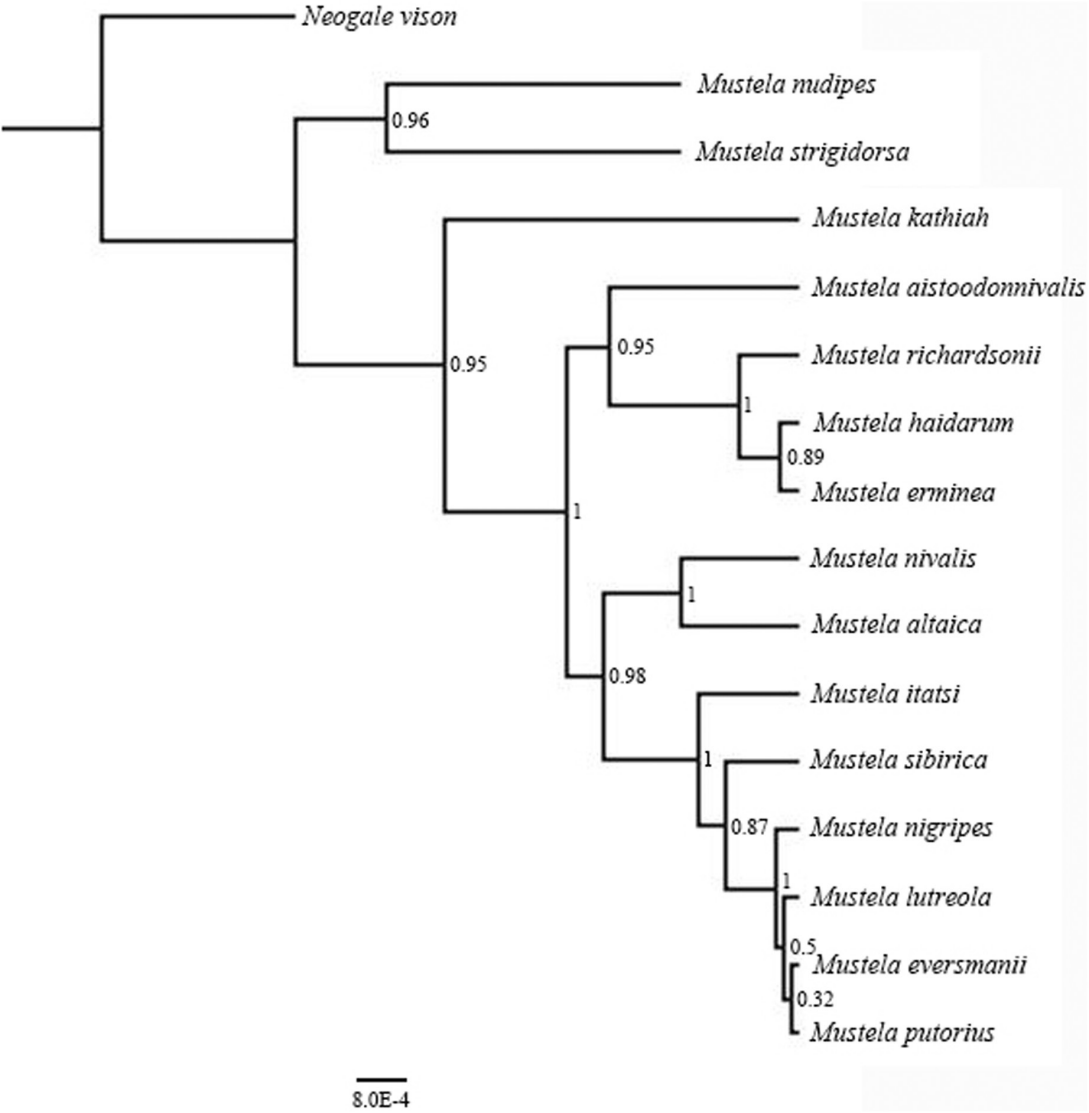
Species delimitation mtDNA+nDNA gene species trees using the BEAST model. The phylogenetic tree includes all species of genus *Mustela* and ends with the species name. Numbers at nodes refer to Bayesian posterior probabilities. Scale bars represent substitutions per site.

The topology of the *BEAST species tree differed slightly from those of the mitochondrial and nuclear gene trees (Figure 5). The position of *M. aistoodonivalis* was the same as in the mitochondrial and nuclear gene trees with strong support (pp = 0.95). *Mustela richardsonii*, *M. nigripes*, *M. nivalis*, *M. altaica*, and *M. lutreola* also obtained high support in the *BEAST species tree (pp = 1).

The Kimura‐2‐parameter (K2p) distances between the species of *Mustela* ranged from 0.008 to 0.158 (Table 5). The average K2p between *M. aistoodonnivalis* and its congeners was 0.094, and between *M. nivalis* and *M. aistoodonnivalis* K2p = 0.082. The K2p of *M. erminea*, *M. haidarum*, and *M. richardsonii* with *M. aistoodonnivalis* was 0.062, 0.071, and 0.073, respectively.

**TABLE 5:** Kimura‐2‐parameter distances between *Mustela* species based on the *CYTB* gene.

No.	Species	1	2	3	4	5	6	7	8	9	10	11	12	13	14
1	*M. aistoodonnivalis*														
2	*M. altaica*	0.085													
3	*M. erminea*	0.062	0.065												
4	*M. eversmanii*	0.071	0.087	0.079											
5	*M. haidarum*	0.071	0.075	0.012	0.085										
6	*M. itatsi*	0.074	0.103	0.077	0.055	0.085									
7	*M. kathiah*	0.085	0.107	0.086	0.108	0.091	0.099								
8	*M. lutreola*	0.074	0.086	0.080	0.008	0.088	0.055	0.111							
9	*M. nigripes*	0.077	0.092	0.083	0.026	0.090	0.058	0.111	0.026						
10	*M. nivalis*	0.082	0.072	0.075	0.083	0.079	0.086	0.101	0.083	0.086					
11	*M. nudipes*	0.132	0.150	0.128	0.158	0.133	0.149	0.122	0.155	0.158	0.155				
12	*M. richardsonii*	0.073	0.078	0.022	0.086	0.033	0.080	0.101	0.088	0.091	0.084	0.128			
13	*M. sibirica*	0.068	0.083	0.075	0.034	0.086	0.047	0.098	0.031	0.037	0.079	0.146	0.078		
14	*M. strigidorsa*	0.132	0.144	0.122	0.141	0.124	0.138	0.123	0.138	0.145	0.123	0.115	0.127	0.136	
15	*M. putorius*	0.075	0.082	0.082	0.011	0.088	0.059	0.110	0.008	0.028	0.083	0.153	0.086	0.033	0.137

### 3.4 Morphological analysis

Morphological index measurement data are shown in Table 6, and complete measurement data are provided in Table S2.

**TABLE 6:** Linear measurement data of *Mustela* species skulls.

Measurement	*M. altaica*	*M. aistoodonnivalis*	*M. erminea*	*M. eversmanii*	*M. kathiah*	*M. nivalis*	*M. sibirica*	*M. strigidorsa*
	*n* = 4	*n* = 6	*n* = 5	*n* = 6	*n* = 7	*n* = 5	*n* = 21	*n* = 5
Profile length	40.28 ± 2.83	31.36 ± 1.82	52.91 ± 6.28	67.76 ± 4.39	48.81 ± 1.49	32.64 ± 1.84	56.66 ± 6.27	57.13 ± 4.01
	(36.54–43.41)	(29.79–34.16)	(45.91–60.78)	(61.44–72.23)	(46.40–49.95)	(30.20–35.17)	(48.03–66.68)	(52.09–60.91)
Basal length	37.28 ± 2.65	29.2 ± 1.6	51.91 ± 6.26	65.49 ± 2.98	45.82 ± 1.14	30.46 ± 1.78	52.97 ± 5.64	54.45 ± 3.03
	(33.72–39.85)	(27.42–31.73)	(44.35–60.03)	(61.58–68.55)	(44.12–47.12)	(27.85–32.73)	(45.76–62.89)	(51.95–58.22)
Median palatal length	16.93 ± 1.66	12.27 ± 0.87	24.52 ± 4.39	34.37 ± 2.15	21.27 ± 0.78	13.24 ± 0.94	25.43 ± 3.04	27.04 ± 2.21
	(14.76–18.81)	(11.48–13.64)	(20.22–31.13)	(31.35–37.13)	(19.84–22.22)	(11.58–13.87)	(21.98–30.87)	(24.11–29.61)
Least breadth between the orbits	7.7 ± 1.01	6.53 ± 0.27	12.98 ± 2.22	18.63 ± 2.22	9.84 ± 1.04	6.86 ± 0.96	11.33 ± 1.50	13.67 ± 1.48
	(6.67–9.06)	(6.29–6.93)	(10.92–16.28)	(16.47–23.42)	(7.84–10.75)	(5.34–7.69)	(9.71–14.09)	(11.91–15.71)
Least breadth behand the postorbital process	8.93 ± 0.83	7.78 ± 0.77	12.64 ± 2.31	14.28 ± 1.14	11.04 ± 1.51	7.37 ± 0.54	11.61 ± 1.31	14.59 ± 1.39
	(7.90–9.94)	(6.51–8.76)	(10.23–16.16)	(12.7–15.88)	(9.40–13.34)	(7.01–8.28)	(8.42–13.38)	(12.88–15.89)
Zygomatic breadth	19.19 ± 2.94	16.12 ± 0.83	30.66 ± 4.16	40.31 ± 3.93	25.09 ± 1.15	16.79 ± 0.91	29.28 ± 4.77	32.74 ± 3.02
	(16.49–22.32)	(14.83–17.28)	(26.32–37.17)	(33.32–45.49)	(23.45–26.56)	(15.96–18.07)	(23.08–37.80)	(29.71–36.83)
Length of tympanic bulla	12.17 ± 0.78	9.75 ± 0.57	16.17 ± 1.11	17.79 ± 1.86	14.61 ± 0.36	10.95 ± 0.93	16.75 ± 1.39	15.86 ± 0.97
	(11.03–12.68)	(9.01–10.36)	(14.81–17.66)	(14.89–21.14)	(14.25–15.15)	(10.12–12.53)	(14.76–19.62)	(14.88–17.13)
Greatest width of the tympanic bulla	7.16 ± 0.67	6.17 ± 0.35	10.61 ± 1.72	13.67 ± 2.33	7.01 ± 0.52	6.23 ± 0.57	9.59 ± 1.72	9.99 ± 0.72
	(6.34–7.98)	(5.60–6.53)	(8.89–13.13)	(11.33–17.58)	(6.54–7.71)	(5.33–6.86)	(7.19–13.10)	(8.71–10.48)
Greatest neurocranium breadth	19.05 ± 1.64	15.22 ± 1.17	27.50 ± 4.83	30.98 ± 2.47	21.31 ± 1.26	15.41 ± 0.42	24.09 ± 2.07	26.53 ± 1.63
	(17.78–21.27)	(13.73–16.52)	(21.85–33.09)	(27.92–35.17)	(20.47–23.86)	(15.13–16.02)	(20.93–27.53)	(23.96–28.48)
Height of the braincase	15.74 ± 1.68	11.52 ± 0.93	24.81 ± 0.42	27.21 ± 2.49	16.5 ± 0.50	12.57 ± 0.41	21.75 ± 2.12	20.35 ± 2.05
	(13.38–16.99)	(10.50–12.78)	(24.55–25.44)	(24.62–30.65)	(15.64–17.01)	(11.93–13.05)	(18.67–25.57)	(18.02–22.59)
Oral length of the vertical ramus	20.41 ± 2.28	15.52 ± 0.8	29.51 ± 5.37	43.07 ± 2.75	26.68 ± 1.09	16.19 ± 1.16	31.37 ± 3.99	35.05 ± 2.87
	(17.60–23.15)	(14.53–16.71)	(25.02–37.08)	(38.33–45.56)	(24.7–27.88)	(14.72–17.86)	(26.77–38.41)	(31.63–38.06)
Oral height of the vertical ramus	9.24 ± 1.11	7.47 ± 0.62	14.64 ± 3.33	20.44 ± 1.49	13.17 ± 0.74	7.79 ± 0.40	15.51 ± 2.63	17.41 ± 1.58
	(8.45–10.88)	(6.84–8.28)	(11.64–19.37)	(18.05–22.12)	(12.01–14.07)	(7.39–8.35)	(12.79–20.63)	(15.28–18.6)
Length between incisor and occipital condyles	40.13 ± 2.89	31.53 ± 1.74	55.49 ± 6.31	69.96 ± 3.11	49.01 ± 0.98	32.82 ± 1.87	56.77 ± 5.95	57.21 ± 4.35
	(36.19–42.97)	(29.76–34.32)	(48.49–63.95)	(65.61–72.56)	(47.62–50.31)	(30.31–35.45)	(49.16–67.40)	(52.18–62.31)

Bartlett's test rejected the null hypothesis (*χ* ^2^ = 1703.319, *p* = .000), which meant the data were spherical and the variables were somewhat independent of each other. KMO was 0.913, indicating that there was a strong correlation among the variable data of skull morphology, which was suitable for factor analysis. Two principal components, explaining 92.506% of the morphological variation, were extracted from the analysis. Its factor loading values were all positive and most were greater than 0.95 (Table 7), indicating that it was mainly related to the overall size of the skull.

**TABLE 7:** Character loadings, eigenvalues, and proportion of variance explained by the first two axes (PC 1 and PC 2) of a principal component analysis using the log_10_‐transformed measurements of *Mustela*.

Measurement	Principal componen	
	1	2
Median palatal length (MPL)	0.991	−0.057
Basal length (BL)	0.991	−0.099
Oral length of the vertical ramus (OLVR)	0.990	0.017
Length between incisor and occipital condyles (LIOC)	0.990	−0.110
Zygomatic breadth (ZB)	0.989	0.037
Profile length (PL)	0.986	−0.098
Oral height of the vertical ramus (OHVR)	0.986	−0.006
Least breadth between the orbits (LBO)	0.963	0.159
Greatest neurocranium breadth (GNB)	0.960	0.124
Height of the braincase (HB)	0.940	−0.124
Length of tympanic bulla (LTB)	0.929	−0.290
Greatest width of the tympanic bulla (GWTB)	0.921	0.067
Least breadth behand the postorbital process (LBPP)	0.858	0.427
Eigenvalue	12.493	0.045
Variance explained	92.506	2.793

*Note*: The meanings of variable abbreviations are given in the Section 2 (Materials and Methods) section.

**FIGURE 6:**
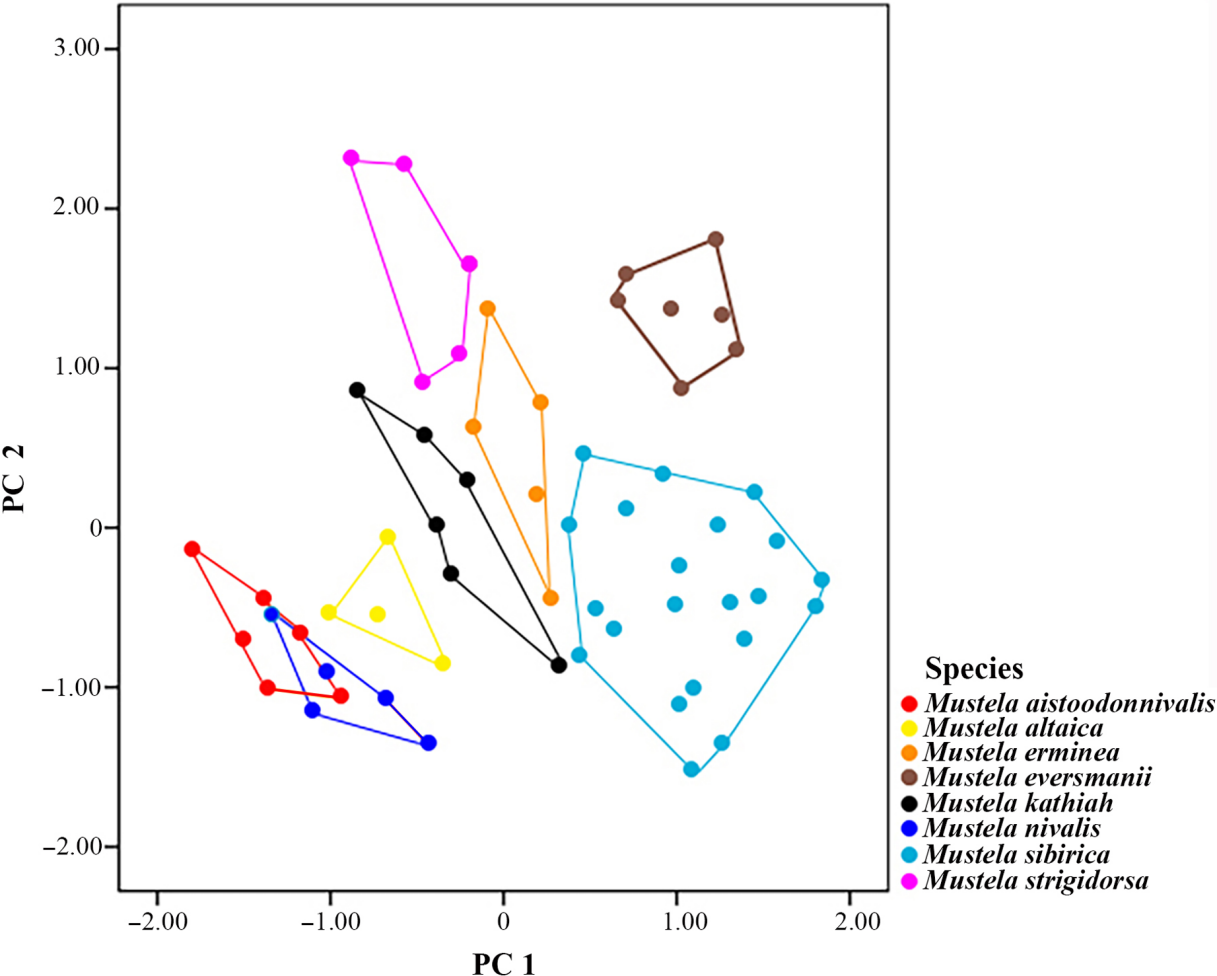
Scatterplot of principal component analysis based on linear measurements of eight species of *Mustela* distributed in China. The numbers on the horizontal axis represent REGR factor score 1 for Analysis 1. The numbers on the vertical axis represent REGR factor score 2 for Analysis 1.

Using PC 1 and PC 2 maps (Figure 6), the larger *M. strigidorsa* plotted in the negative region of PC 1 and the positive region of PC 2. *Mustela eversmanii* plotted in the positive region of PC 1 and PC 2. *Mustela erminea* and *M. kathiah* mostly concentrated in the positive region of PC 2 and were clearly distinguished. The smaller *M. altaica* was clearly distinguished from the other two species, while there was some overlap between *M. aistoodonnivalis* and *M. nivalis*.

### 3.5 Geometric morphometric analyses

**FIGURE 7:**
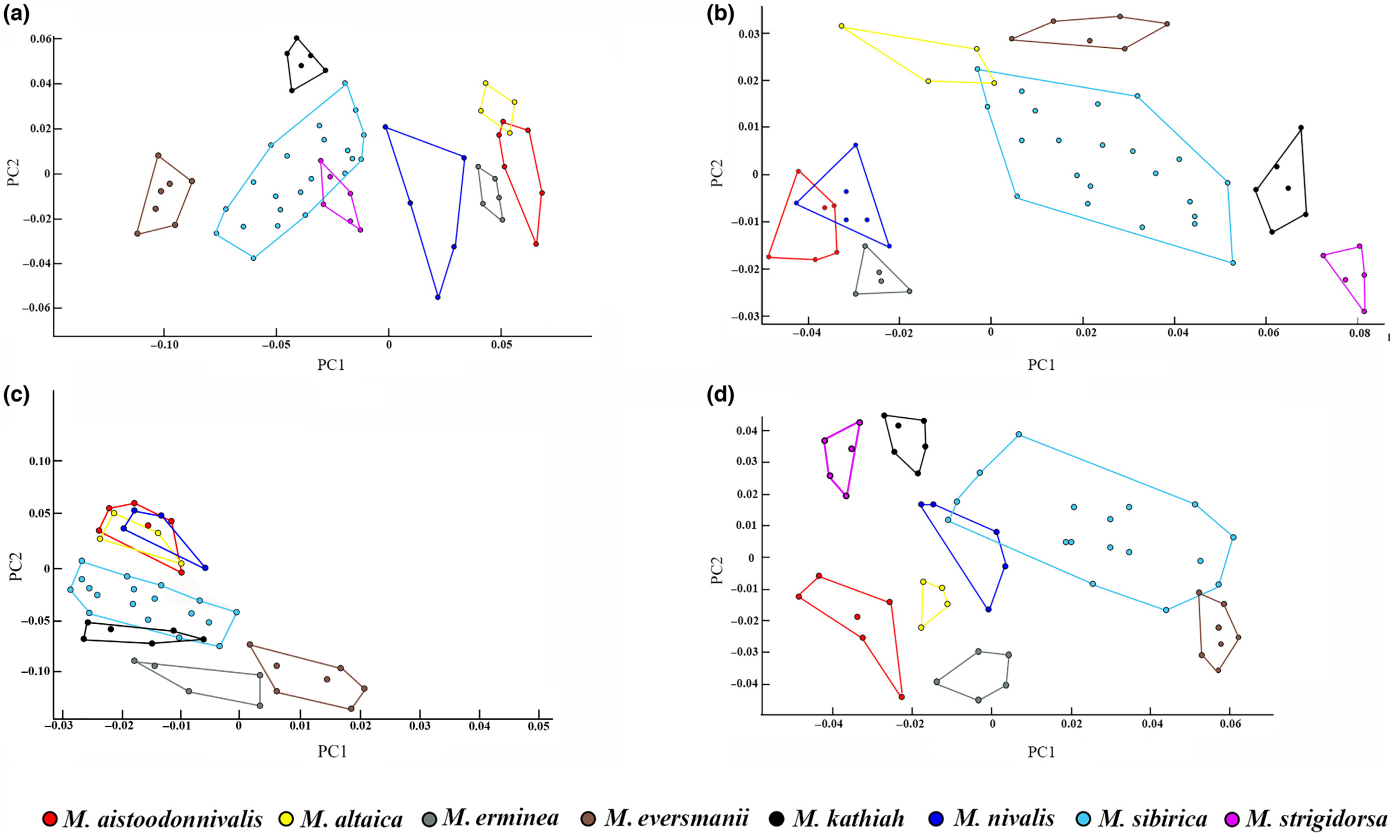
Scatterplot of principal component analysis based on skull of eight species of *Mustela* distributed in China referred from landmarks/semi‐landmarks. The numbers on the horizontal axis represent REGR factor score 1 for Analysis 1. The numbers on the vertical axis represent REGR factor score 2 for Analysis 1.

The analysis from the ventral view of the cranium (Figure 7a; Figure S4(A)) showed several morphological differences among the species of *Mustela*. *Mustela nivalis* and *M. aistoodonnivalis* were clearly distinguished. *Mustela aistoodonnivalis* and *M. altaica* showed a minor overlap. *Mustela sibirica* and *M. strigidorsa* showed a larger overlap. *Mustela erminea*, *M. kathiah*, and *M. eversmanii* were clearly separated from each other and the remaining species.

The analysis of the dorsal views of the cranium (Figure 7b; Figure S4(B)) showed a substantial overlap of *M. aistoodonnivalis* and *M. nivalis*. *Mustela altaica*, and *M. sibirica* overlapped partially. *Mustela erminea*, *M. eversmanii*, *M. kathiah*, and *M. strigidorsa* were clearly separated from each other and other species.

The analysis of the lateral view of the cranium (Figure 7c; Figure S4(C)) could not distinguish *M. altaica*, *M. aistoodonnivalis*, and *M. nivalis* because they more or less occupied the same area. *Mustela kathiah, M. sibirica*, and *M. strigidorsa* showed an even larger percentage of overlap. *Mustela erminea* and *M. eversmanii* were well distinguished from other species.

The data for the lateral view of the mandible (Figure 7d; Figure S4(D)) showed that *M. aistoodonnivalis* was well‐differentiated from other species; it did not overlap with *M. altaica* or *M. nivalis*. *M. erminea, M. eversmanii, M. kathiah*, and *M. strigidorsa* were well separated. *Mustela eversmanii, M. sibirica*, and *M. nivalis* overlapped partially.

In short, our molecular and morphological comparisons supported *M. aistoodonnivalis* as a valid species.

**Detailed redescription of *Mustela aistoodonnivalis* (**Wu & Kao, 1991 **)**

缺齿伶鼬 Que Chi Ling You (Lacked‐teeth pygmy weasel).

Measurements. W: 39–48 g; HBL: 124–155 mm; TL: 58–70 mm; HFL: 20–25 mm; EL: 10–12 mm.

Type locality: Taibai Mountains, Shaanxi, China, 2750 m above sea level.

Holotype specimen: Herbarium of the Shaanxi Zoological Research Institute, Collection No. 84009. Female.

Diagnosis: A smaller species in the genus *Mustela*, with a body shape similar to *M. nivalis* and distinguished from other species of *Mustela* by the following characteristics: tail long, about half the head–body length; back and end of tail dark brown; white throat and light yellowish abdomen tinged with rusty red; forelimb fur brown; sagittal suture faint or almost lacking; mandible slender; and second lower molar lacking.

Description: *M. aistoodonnivalis* is a *Mustela* species with smaller individuals, weighing 39–48 g, body length 124–155 mm, and tail length 58–70 mm. The hindfoot length is 20–25 mm, and ear length 10–12 mm. The general appearance is similar to *M. nivalis*; however, the tail length is more than 1/3 the head–body length, and summer dorsal hairs are dark brown from the upper lip edge through the cheeks backward, as well as along the side of the body to the tail and the outside of the limbs. The ears are dark brown, covered with short hairs of the same color. The ventral part of the chin to the throat is white. The neck to the abdomen is yellowish, tinged with rusty red. Individual specimens have less obvious rusty red patches, and the dorsal and ventral boundaries are clear. The dorsal surfaces of the front and hindfeet are slightly lighter in color, with some individuals having white medial forefeet and a few white hairs covering the dorsal surfaces of the carpal palps and the dorsal surfaces of the hind toes. Pelage exhibits no seasonal variation; a specimen collected in beginning of November (SAF181732) had the same pelage color as in specimens collected in summer.

The dorsal surface of the skull is relatively flat, the cerebral skull is broad, and the muzzle is narrow and short and significantly recurved. The interorbital width is quite large, and there is a less pronounced postorbital protrusion. The bony sutures between the nasal bone, frontal bone, parietal bone, interparietal bone, and squamous bone are healed and poorly defined. The occipital foramen magnum is broadly rounded and oval, with a small occipital condyle. On the lateral side of the skull, the anterior orbital foramen is obvious (located below the slender anterior margin of the orbit) and the zygomatic arch is slender and curved upward. At the ventral surface of the skull, the incisor foramen is very short, and the hard palate is triangular in shape and consists of the maxilla and premaxilla. The basioccipital bone is almost fused with the anterior pterygoid and basi‐pterygoid, with indistinct sutures and a narrow rectangular shape. The auditory vesicle is large and full, long and oval, with a flat, nearly parallel inner margin.

The tooth pattern is 3.1.3.1/3.1.3.1 = 32. The upper incisors are transverse, the third incisor is thicker, flatter, and inclined backward. The canines are longer than the incisors, slightly curved, and twice as tall as the third incisor. The first and second premolars are tricuspid, with the first premolar having an inconspicuous anterior cusp and a small protocusp and being slightly higher than the canine root. The second premolar has a more pronounced protocusp, orthogonally oriented upward, with a more rounded anterior and posterior cusp. The third premolars are “Y”‐shaped, with the inner lobe of the anterior margin larger than the outer lobe. The original cusps are broad and sharp. The upper molars are transverse, “dumbbel” shaped, with the inner lobe rounded and flattened with a small cusp and the outer lobe with two small cusps. The mandibular incisors are arranged in an inverted zigzag pattern, with the second incisor located behind the first and third incisors. The canine teeth are stubby and more curved, shorter than the maxillary canines. The first and second premolars are both unicuspid. The third premolar has three cusps, with the primary cusp being more pronounced and much larger than the first and second premolars. The lower molars consist of three lobes in a “W” shape, which forms a well‐developed cleft tooth with the maxillary third premolars. There is no second lower molar.

**FIGURE 8:**
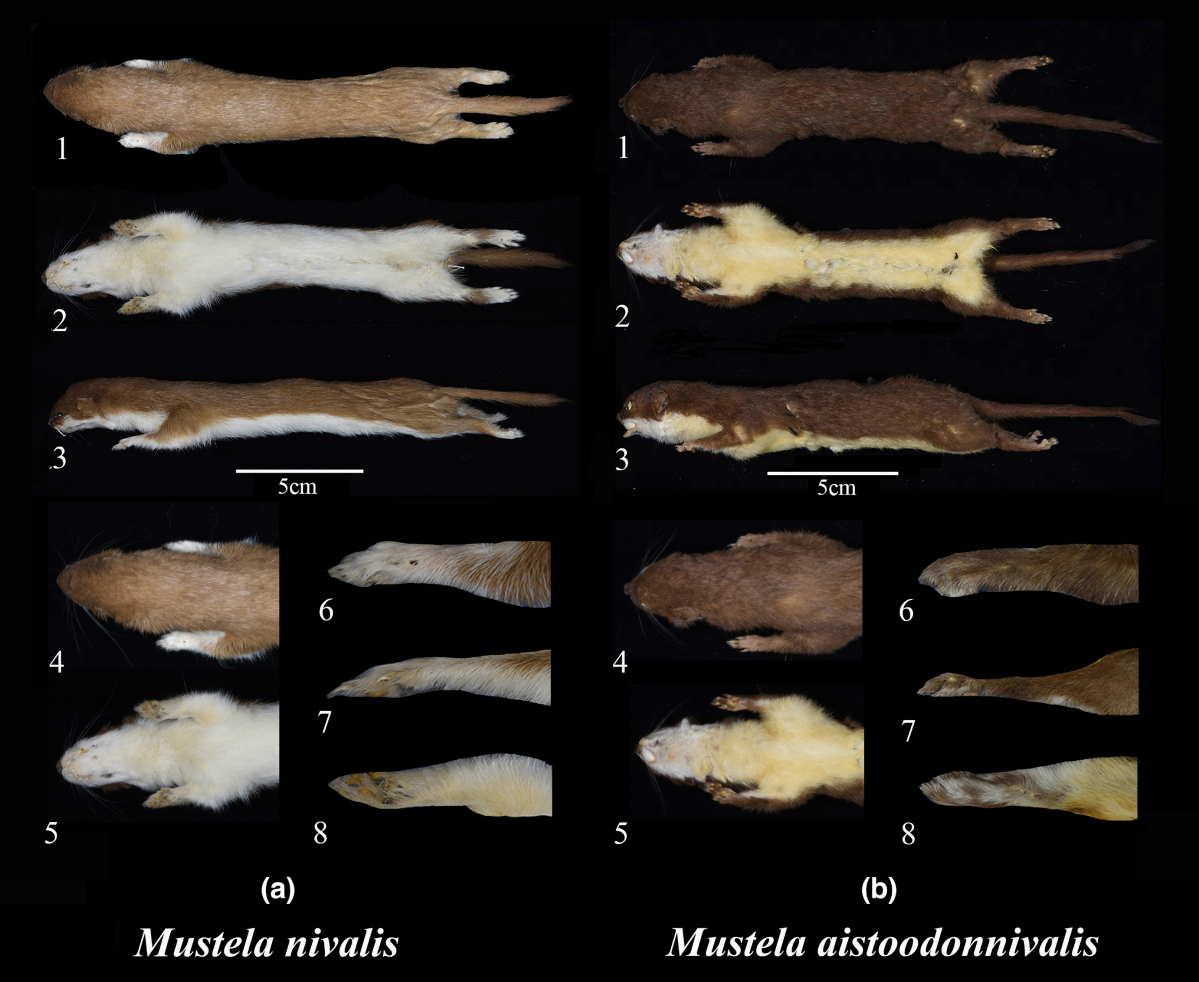
Comparison between *Mustela aistoodonnivalis* (voucher number: SAF191315) and *Mustela nivalis* (SAF17440). 1: color of the dorsal view; 2: color of the ventral view; 3: color of the lateral view; 4: color of head and fore limb; 5: color of throat; 6: color of the dorsal view of the fore limb; 7: color of the lateral view of the fore limb; and 8: color of the ventral view of the fore limb.

Comparison: *M. aistoodonnivalis* is similar in body shape to *M. nivalis*. *Mustela nivalis* is brown to dark brown on the back and tail end, and *M. aistoodonnivalis* is dark brown (Figure 8a1,b1; Table 8; Figure S1). Ventrally, *M. nivalis* is white or pale yellow from throat to abdomen and *M. aistoodonnivalis* has a white throat and yellowish abdomen tinged with rusty red (Figure 8a2‐a4,b2‐b4; Table 8; Figure S1). Forelimb fur of *M. aistoodonnivalis* is brown, with only some white fur covering the digitals, whereas in *M. nivalis*, all fur is white (Figure 8a5‐a8,b5‐b8; Table 8; Figure S1).

**TABLE 8:** Morphological comparison of *Mustela aistoodonnivalis* and *M. nivalis.*

Structure		*M. aistoodonnivalis* (*n* = 6)	*M. nivalis* (*n* = 5)
Coat color	Dorsum	Dark brown (summer)	Light brown‐red (summer) or white (winter)
	Abdomen	Light yellow	White or pale
	Tail	Dark brown around tail	Light brown
	Forelimb	Dark brown	White
Appearance	HBL	150 mm (127.8–165.2 mm)	170 mm (157–183 mm)
	TL	70 mm (50–62 mm)	55 mm (20–53 mm)
	TL/HBL	46.7%	32.4%
Skull	Profile length	30.49 mm (29.79–34.16 mm)	33.47 mm (32.12–35.17 mm)
	Zygomatic breadth	15.65 mm (14.83–16.53 mm)	16.77 mm (16.37–18.07 mm)
	M_2_	Missing	Existing
	Dental formula	3.1.3.1/2.1.3.1 = 32	3.1.3.1/3.1.3.2 = 34

**FIGURE 9:**
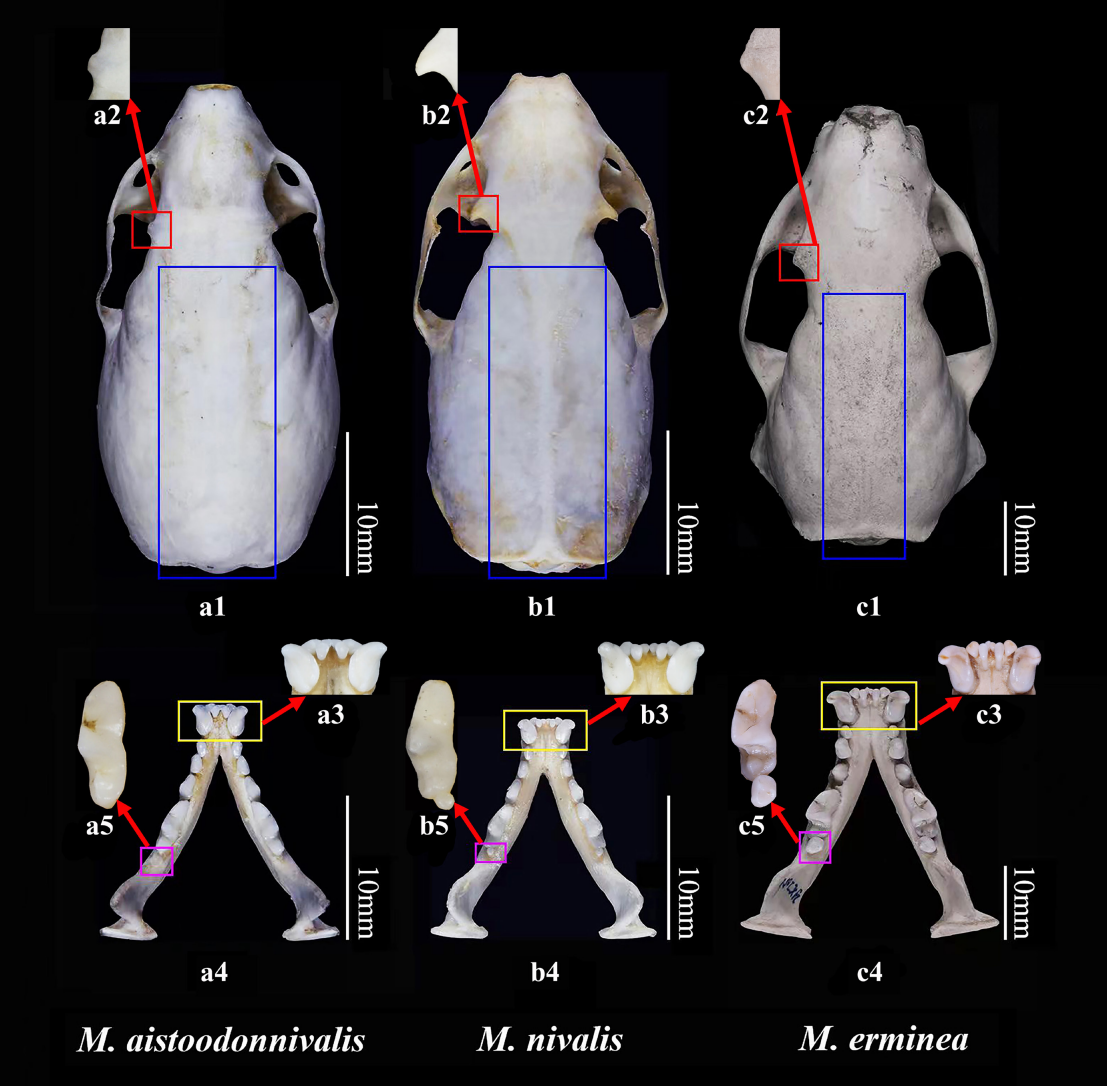
Comparison of the cranium and the mandible among *Mustela aistoodonnivalis* (voucher number: SAF181732), *Mustela erminea* (YX2051) and *Mustela nivalis* (SAF170306). (a1) the dorsal view of the cranium of *M. aistoodonnivalis* (sagittal suture absent); (a2) the enlarged postorbital process of *M. aistoodonnivalis* (short and inconspicuous); (a3) the enlarged mandibular incisors of *M. aistoodonnivalis* (the distal of the second lower incisor protrudes inward and hook‐shaped); (a4) the mandible of *M. aistoodonnivalis* (slender); (a5) the enlarged lower molar of *M. aistoodonnivalis* (only one lower molar); (b1) the dorsal view of the cranium of *M. nivalis*(sagittal suture distinct); (b2) the enlarged postorbital process of *M. nivalis* (obvious); (b3) the enlarged mandibular incisors of *M. nivalis* (the distal of the second lower incisor flush with the first and third incisors); (b4) the mandible of *M. nivalis* (thick); (b5) the enlarged lower molars of *M. nivalis* (two lower molar); (c1) the dorsal view of the cranium of *M. erminea* (sagittal suture absent); (c2) the enlarged postorbital process of *M. erminea* (obvious); (c3) the enlarged mandibular incisors of *M. erminea* (the distal of the second lower incisor flush with the first and third incisors); (c4) the mandible of *M. erminea* (thick); (c5) the enlarged lower molars of *M. erminea* (two lower molar). Red square shows the postorbital process, blue square shows the sagittal suture, yellow square shows the incisors, and purple square shows the lower molars.

The skull of *M. nivalis* has a distinct sagittal suture that is absent in *M. aistoodonnivalis* and *M. erminea* (Figure 9a1‐c1). The postorbital process of *M. nivalis* and *M. erminea* is obvious, but short and inconspicuous in *M. aistoodonnivalis* (Figure 9a2‐c2). Further, the distal of the second lower incisor of *M. nivalis* and *M. erminea* is flush with the first and third incisors (Figure 9b3,c3), but in *M. aistoodonnivalis* it protrudes inward and is hook‐shaped (Figure 9a3). The mandible of *M. nivalis* and *M. erminea* is thick (Figure 9b4,c4), while the mandible of *M. aistoodonnivalis* is slender (Figure 9a4). *Mustela nivalis* and *M. erminea* have a second lower molar, which is missing in *M. aistoodonnivalis* (Figure 9a5‐c5; Figure S1).

Distribution: Currently, the species is known in the Qinling Mountains of southern Shaanxi and northern Sichuan, including Pingwu, Li County, Jiuzhai, Baoxing, and Heishui.

Habitat: It mainly inhabits coniferous forests and meadows between 1900 and 2880 m above sea level, mostly in slightly moist habitats. Specimens for this study were collected mainly from alpine scrub and grassland junction zones, from secondary mixed coniferous forests, broad‐leaved scrub, and weedy areas.

## 4 DISCUSSION

Wu and Kao (1991) considered *M. nivalis* to be a close relative of *M. aistoodonnivalis*, but our phylogenetic study demonstrates that *M. aistoodonnivalis* constitutes a well‐defined clade and does not cluster in the same clade with *M. nivalis*. The genetic distances calculated based on the *CYTB* also indicate that *M. aistoodonnivalis* differs from its congeners. Morphological differences between *M. aistoodonnivalis* and its congeners are evident and include the absence of the second lower molar, a light yellow throat and abdomen, and a small number of white hairs covering the toes of the forefeet and hindfeet. In summary, we reconfirm *M. aistoodonnivalis* as an independent species.

In Sichuan, Kingding (Tatsienlu) is the type locality of *M. nivalis russelliana*, which was described by Thomas (1911). We failed to collect specimens of *M. nivalis russelliana* because of its rarity. It was documented to occur only in the northwestern Sichuan plateau, including Kangding, Luhuo, Xinlong, Baiyu, Dege, and Ganzi (Allen, 1938; Wang, 2003; Wang & Hu, 1984; Wang & Hu, 1999). According to original description, upper surface of *M. nivalis russelliana* is uniform dark brown and the under surface a beautiful pinkish buff, turning into white anteriorly on the chin, interramia, and lips. A dark rictal spot is also present and the very sharp line of demarcation runs from the upper lip to ankle. Its tail is uniformly brown and no tuft occurs. From appearance, it differs from *M. aistoodonnivalis* (see description of *M. aistoodonnivalis*). On measurements, *M. aistoodonnivalis* has longer tail length. The longest tail of the type series of *M. nivalis russelliana* is 54 mm, about 40% of head–body length, but in *M. aistoodonnivalis*, the shortest tail length is 58 mm (average: 63 mm), approximately 46% of head–body length. A *t*‐test using data for *M. nivalis russelliana* from original description Thomas (1911) and Gao (1987) obtains a significant difference (*p* = .004 < .05). Thus, we reject the hypothesis that *M. aistoodonnivalis* and *M. nivalis russelliana* are the same taxa.

**FIGURE 10:**
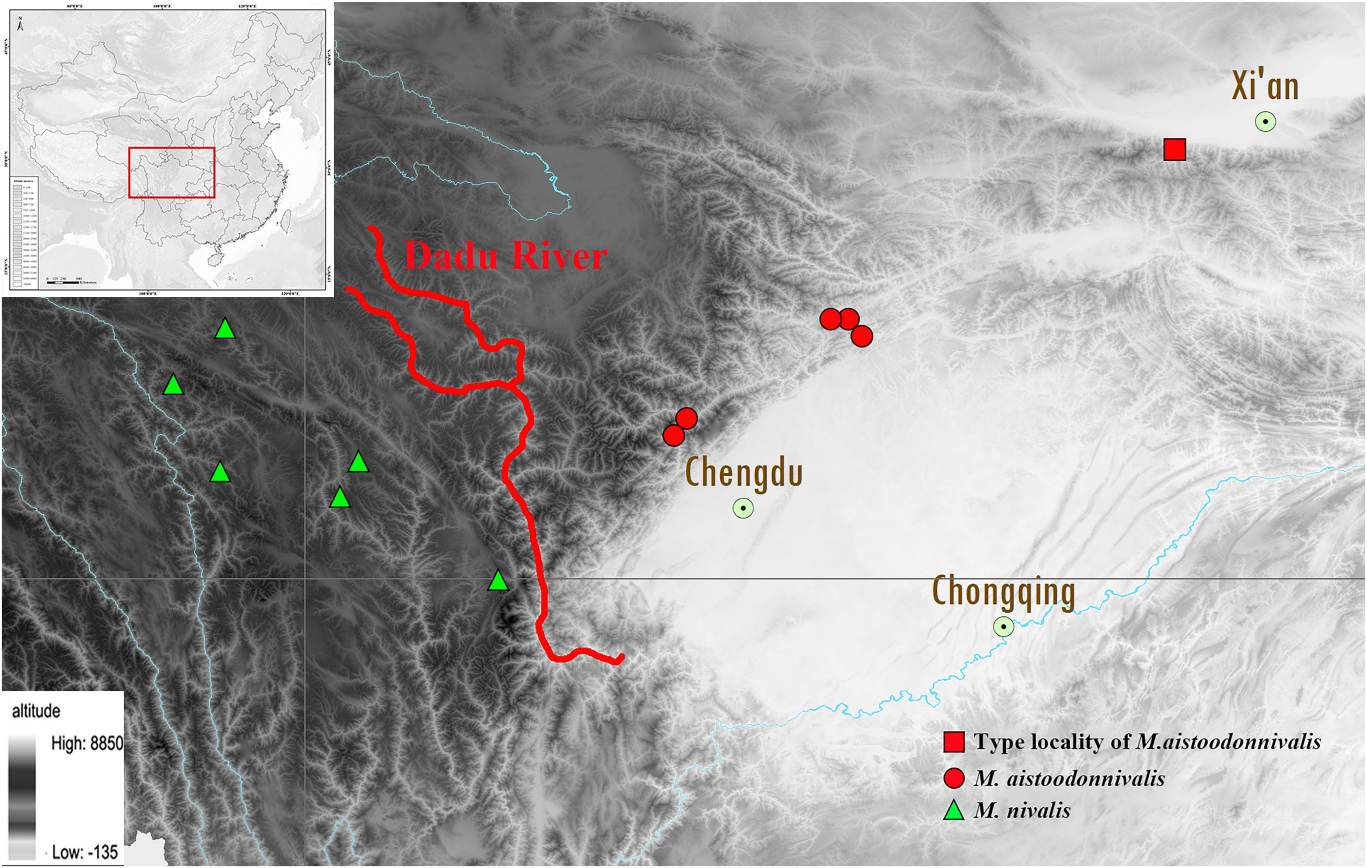
Distribution pattern of *Mustela nivalis* and *M. aistoodonnivalis* in Sichuan province and adjacent region. The intensity of gray in the background represents elevation. Red square in the upper left map indicates the distribution area. The small map at the top left indicates the location of the distribution areas of the two species in China.

On distribution, *M. aistoodonnivalis* mainly occurs in the Qinling Mountains of Shaanxi and in the Minshan and Qionglai mountains of Sichuan, including Pingwu, Jiuzhai, Huanglong, Baoxing, and Heishui (Figure 10). Therefore, we assume that the Dadu River separates *M. nivalis* to the west and *M. aistoodonnivalis* to the east. Comparisons show that *M. aistoodonnivalis* is morphologically similar to species of the subgenus *Gale*. However, several distinct differences exist: the dorsal hairs are slightly darker, the ventral hairs are light yellow, the cranium is less narrow and relatively flatter than in the subgenus *Gale*; and the auditory vesicles are shorter and wider than in *Gale*, and the mandibular second molars are missing (Allen, 1938; Gao, 1987; Pan et al., 2007). These results and the phylogenetic analyses place *M. aistoodonnivalis* outside of *Gale*. Hall (1951) pointed out that the absence of second lower molars in *Mustela* is a stable, long‐term genetic phenomenon rather than a species‐specific variation in adaptation to the environment. Therefore, we suggest that the second lower molars are the most distinctive feature distinguishing *M. aistoodonnivalis* from its congeners. Combined with the molecular data, we speculate that *M. aistoodonnivalis* may be a new subgenus of *Mustela*, which needs further study.

Our phylogenetic analyses indicate that *M. kathiah* constitutes a separate clade not associated with other species of *Gale*. It has large genetic distances of 0.107 and 0.101 to *M. nivalis* and to *M. altaica*, respectively. Hosoda et al. (2011) also found these phylogenetic relationships. Therefore, we confirm that *M. kathiah* might also be placed in a separate subgenus. This, however, requires more evidence from molecular analyses and morphological studies.

## 5 CONCLUSION

Based on molecular and morphological analyses, *M. aistoodonnivalis* is recognized as an independent species in the genus *Mustela*, and when compared to its congeners, the absence of the second lower molar is its most important morphological distinguishing character.

## AUTHOR CONTRIBUTIONS

**Yingxun Liu:** Conceptualization (equal); data curation (equal); formal analysis (lead); investigation (equal); methodology (lead); software (lead); validation (equal); visualization (equal); writing – original draft (lead); writing – review and editing (lead). **Yingting Pu:** Conceptualization (equal); data curation (equal); formal analysis (equal); methodology (equal); software (equal); validation (equal); writing – original draft (equal); writing – review and editing (equal). **Shunde Chen:** Data curation (equal); formal analysis (equal); funding acquisition (equal); resources (equal). **Xuming Wang:** Formal analysis (equal); investigation (equal); methodology (equal); validation (equal). **Robert Murphy:** Writing – review and editing (lead). **Xin Wang:** Investigation (equal); methodology (equal); resources (equal). **Rui Liao:** Data curation (equal); investigation (lead); project administration (equal). **Keyi Tang:** Formal analysis (equal); methodology (supporting); software (supporting); visualization (supporting). **Bisong Yue:** Formal analysis (equal); methodology (equal); resources (equal); supervision (equal); validation (equal); writing – review and editing (equal). **Shaoying Liu:** Data curation (equal); formal analysis (lead); investigation (lead); methodology (lead); project administration (equal); resources (lead); supervision (lead); writing – review and editing (lead).

## FUNDING INFORMATION

This research was funded by the National Natural Science Foundation of China (31970399, 31670388).

## CONFLICT OF INTEREST STATEMENT

The authors declare no conflicts of interest.

## Supporting information


Figure S1

Figure S2

Figure S3

Figure S4

Table S1

Table S2

## Data Availability

New DNA sequences in this study were deposited in Genbank (Accession numbers: MT888695‐MT888797, ON730958‐ON731005) (https://www.ncbi.nlm.nih.gov/)
