# Supplementary material for: Revalidation and expanded description of Mustela aistoodonnivalis (Mustelidae: Carnivora) based on a multigene phylogeny and morphology
Source: Ecol Evol. 2023 Apr 18;13(4):e9944. doi: 10.1002/ece3.9944 (PMC10111237; doi:10.1002/ece3.9944)
Supplement: Supplementary file 1 — Figure S1 [file ECE3-13-e9944-s002.pdf]

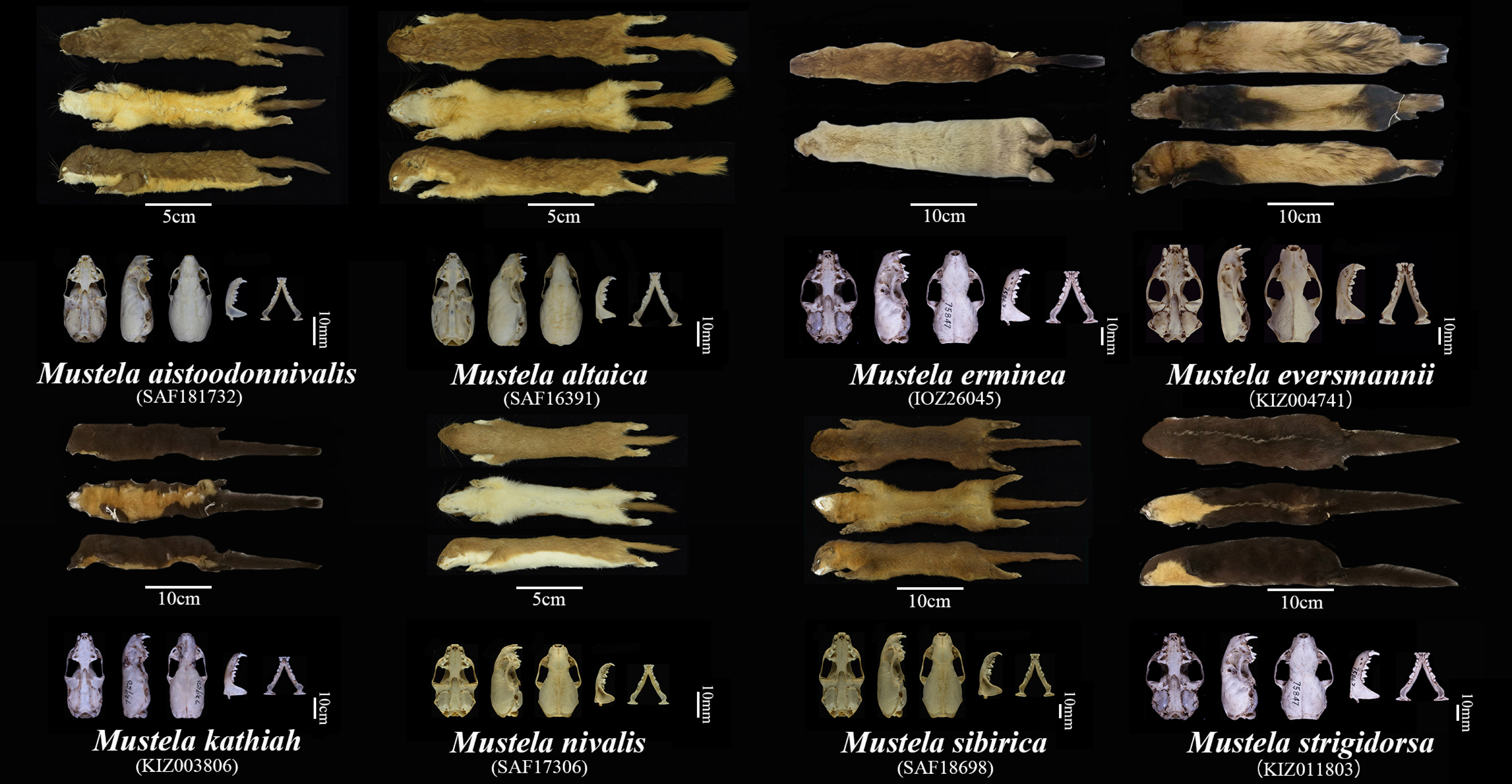
5cm
5cm
10cm
10cm
10mm
10mm
10mm
10mm
Mustela aistoodonnivalis
(SAF181732)
Mustela altaica
(SAF16391)
Mustela erminea
(IOZ26045)
Mustela eversmannii
(KIZ004741)
10cm
5cm
10cm
10cm
10cm
10mm
10mm
10mm
Mustela kathiah
(KIZ003806)
Mustela nivalis
(SAF17306)
Mustela sibirica
(SAF18698)
Mustela strigidorsa
(KIZ011803)
