# Supplementary material for: Revalidation and expanded description of Mustela aistoodonnivalis (Mustelidae: Carnivora) based on a multigene phylogeny and morphology
Source: Ecol Evol. 2023 Apr 18;13(4):e9944. doi: 10.1002/ece3.9944 (PMC10111237; doi:10.1002/ece3.9944)
Supplement: Supplementary file 2 — Figure S2 [file ECE3-13-e9944-s005.pdf]

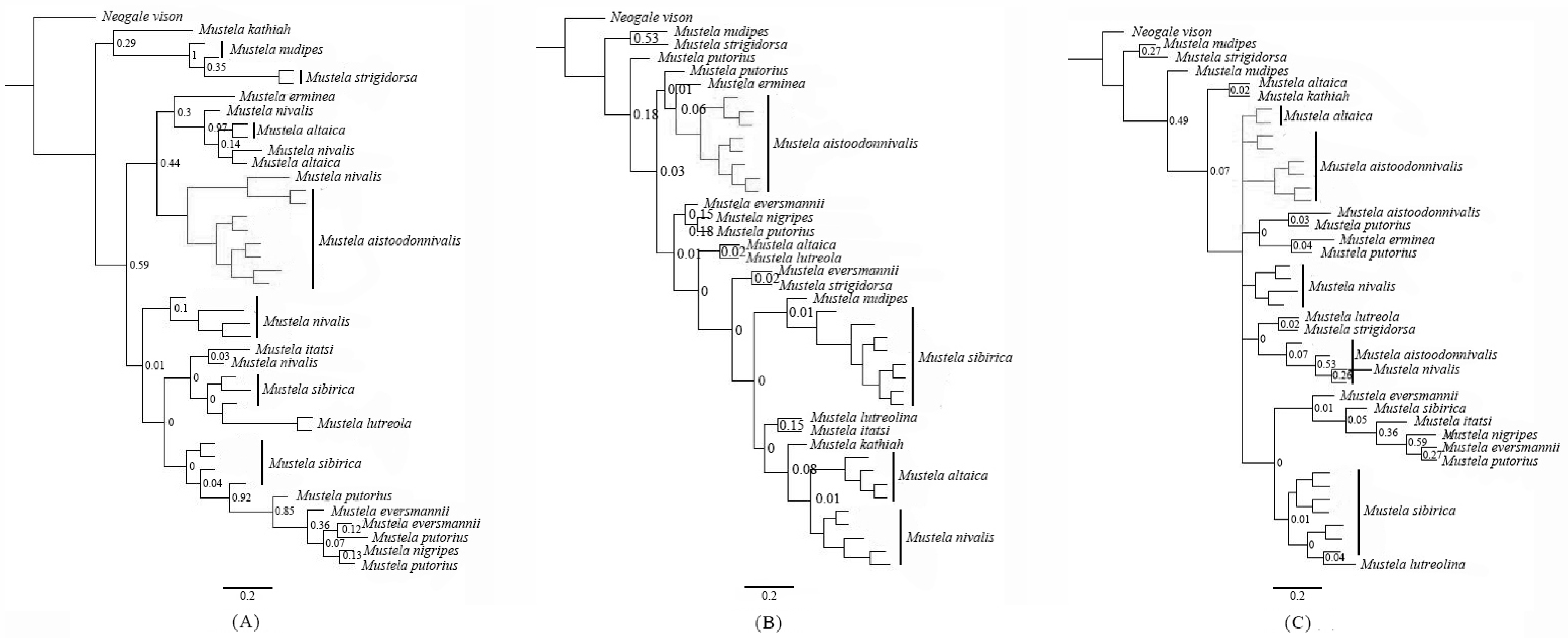

(A) Bayesian phylogenetic analyses based on APOB gene, (B) Bayesian phylogenetic analyses based on ATP7A gene, (C) Bayesian phylogenetic analyses based on BDNF gene. Numbers at nodes refer to Bayesian posterior probabilities. Scale bars represent substitutions per site.
