# Supplementary material for: Revalidation and expanded description of Mustela aistoodonnivalis (Mustelidae: Carnivora) based on a multigene phylogeny and morphology
Source: Ecol Evol. 2023 Apr 18;13(4):e9944. doi: 10.1002/ece3.9944 (PMC10111237; doi:10.1002/ece3.9944)
Supplement: Supplementary file 3 — Figure S3 [file ECE3-13-e9944-s001.pdf]

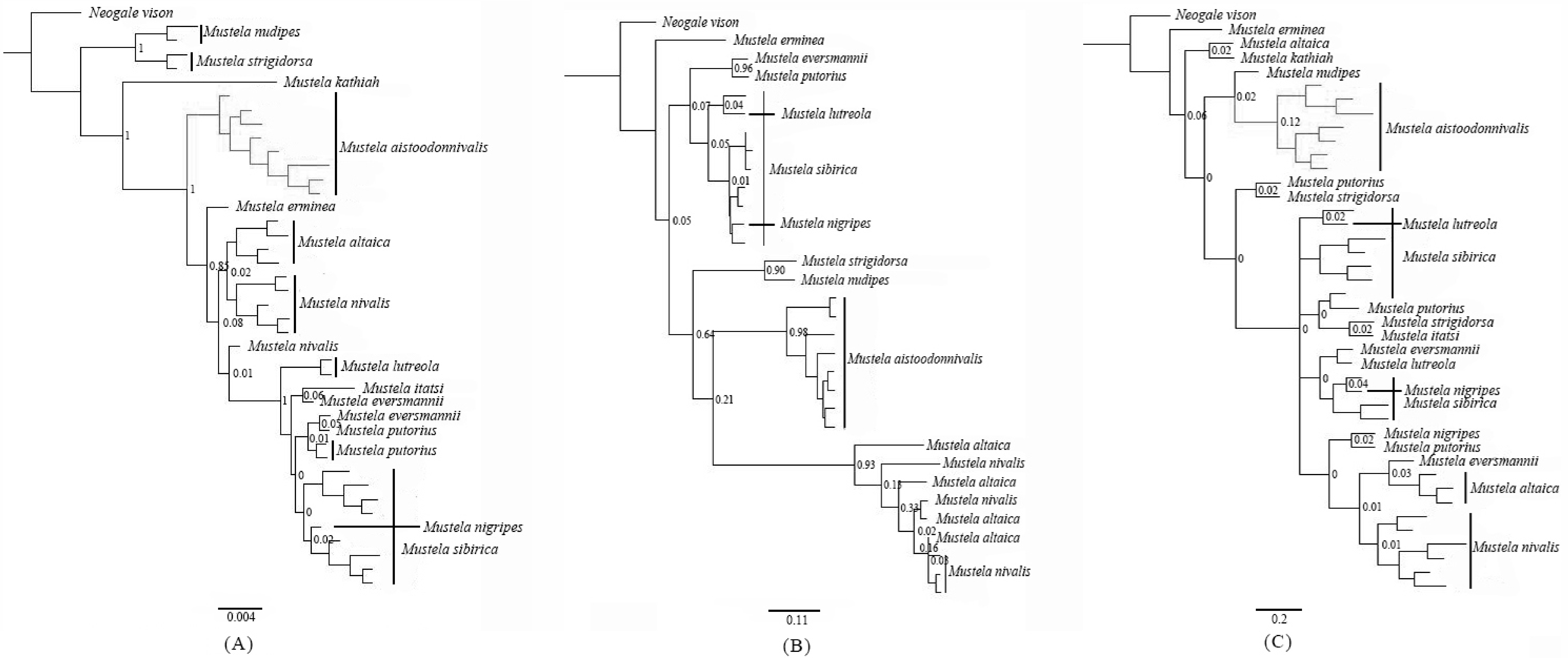

(A) Bayesian phylogenetic analyses based on RAG1 gene, (B) Bayesian phylogenetic analyses based on RAG2 gene, (C) Bayesian phylogenetic analyses based on TMEM20 gene. Numbers at nodes refer to Bayesian posterior probabilities. Scale bars represent substitutions per site.
