# Supplementary material for: Revalidation and expanded description of Mustela aistoodonnivalis (Mustelidae: Carnivora) based on a multigene phylogeny and morphology
Source: Ecol Evol. 2023 Apr 18;13(4):e9944. doi: 10.1002/ece3.9944 (PMC10111237; doi:10.1002/ece3.9944)
Supplement: Supplementary file 4 — Figure S4 [file ECE3-13-e9944-s006.pdf]

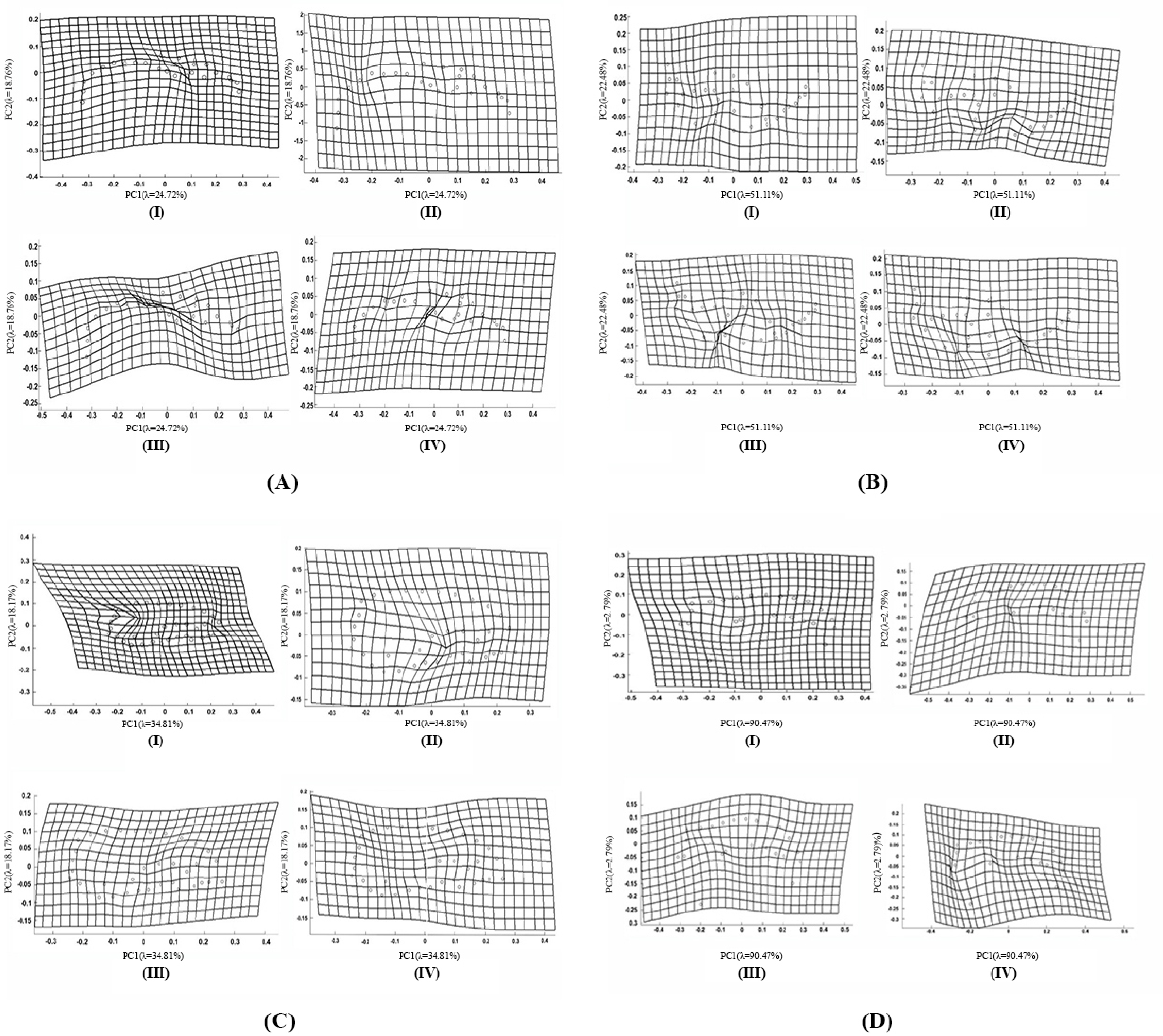

(A) the contour and deformation of the dorsal of the cranium, (B) the contour and deformation of the ventral of the cranium, (C) the contourand deformation of the lateral of the cranium, and (D) the contour and deformation of the mandible.

(I), (II), (III), and (IV) the contour and deformation at the extreme values of PC1, PC2, PC3, and PC4 respectively.

The numbers on the horizontal axis represent REGR factor score 1 for analysis 1.

The numbers on the vertical axis represent REGR factor score 2 for analysis 1.
