# Supplementary material for: Revalidation and expanded description of Mustela aistoodonnivalis (Mustelidae: Carnivora) based on a multigene phylogeny and morphology
Source: Ecol Evol. 2023 Apr 18;13(4):e9944. doi: 10.1002/ece3.9944 (PMC10111237; doi:10.1002/ece3.9944)
Supplement: Supplementary file 5 — Table S1 [file ECE3-13-e9944-s004.docx]

**Table A1. Specimens examined morphologically in this study.**

A total of 59 adult specimens of *Mustela* were examined morphologically in this study. Among those specimens, 29 specimens are housed in the Sichuan Academy of Forestry (SAF). 20 specimens deposited in the Museum of the Kunming Institute of Zoology, Kunming, China (KIZ). two specimens in Institute of Zoology, Chinese Academy of Sciences, Beijing, China (IOZ). three specimens in Xinjiang Uygur Autonomous Region Center for Disease Control and Prevention, Urumchi, China. one specimen from private collection of Kewei Jiang, Urumchi, China, and four specimens in Sichuan University, Chengdu, China. All following numbers are field numbers.

***Mustela aistoodonnivalis*** 6 adult female specimens. csd2000 (Museum No. SAF03190), from Jiuzhaigou county, Sichuan; csd2015 (SAF12703), from Li county, Sichuan; csd2339 (SAF181732), from Pingwu county, Sichuan; csd3528(SAF191315), from Pingwu county, Sichuan; csd3529 (SAF191387), from Jiuzhaigou county, Sichuan; HS-Ⅱ-013(SAF01109), from Songpan county, Sichuan.

***Mustela altaica*** 4 adult female specimens. csd891(SAF17005), from Kangding county, Sichuan; csd2003(SAF16391), from Pulan county, Tibet; csd3531(SAF071101), from Dege county， Sichuan; csd1656(SAF12193), from Barag Youqi county, Nei Mongol.

***Mustela erminea*** 5 adult female specimens. YX2051, from Urumuqi, Xinjiang; 903130568, from Urumuqi, Xinjiang; 0903049, from Barkol Kazakh Autonomous County, Xinjiang; 0903yan198, from Mount Tianshan, Xinjiang; IOZ26045, from Beijing.

***Mustela eversmanii*** 7 adult female specimens. SAF20121, Sichuan; SAF20122, Sichuan; SCDX62, from Seda county, Sichuan; mucuogan02(SAF05680), Ruoergai county, Sichuan;650104 (KIZ004741), from Yushu county, Qinghai; 650105(KIZ004740), from Menyuan, Qinghai. XJ001, Wenquan county, Xinjiang.

***Mustela kathiah*** 6 adult female specimens. SCDX5901, from Mount Emei, Sichuan; 73950(KIZ003806), from Gongshan county, Yunnan; 73601(KIZ003806) from Gongshan county, Yunnan; 030501(KIZ034552), from Yongde county, Yunnan; De134(KIZ034553), from Yongde county, Yunnan; 206812(KIZ016368), from Yongde county, Yunnan.

***Mustela nivalis*** 5 adult female specimens. csd2011(SAF17306), from Hejing county, Xinjiang; csd1480(SCNU01149), from Xinbin county, Liaoning; csd3526(SAF17440), Xinyuan county, Xinjiang; csd3530(SAF19197), from New Barag Right Banner, Nei Mongol; IOZ13325, unknown.

***Mustela sibirica*** 21 adult female specimens. csd3733(SAF19797), from Lijiang, Yunnan; csd2005(SAF18694), from Hanyuan county, Sichuan; csd2006(SAF18695), from Hanyuan county, Sichuan; csd2007(SAF18696), from Hanyuan county, Sichuan; csd2008(SAF18697), from Hanyuan county, Sichuan; csd2379 (SAF18698), from Hanyuan county, Sichuan; HY1(SAF182126), from Hanyuan county, Sichuan; HY2(SAF182127), from Hanyuan county, Sichuan; HY3(SAF182128), from Hanyuan county, Sichuan; CJ01(SAF182129), unknown; CJ30(SAF182130), unknown. SCDX001, from Beijing; SCDX202201, from Chengdu, Sichuan; 820432(KIZ012721), from Kunming, Yunnan; 820133(KIZ010056), from Dege county, Sichuan; 640201(KIZ03046), from Kunming, Yunnan; 88303(KIZ009688), from Wulong, Chongqing; 820199(KIZ012720), from Shiqu county, Sichuan; 631350(KIZ03054), from Anlong county, Guizhou; 631318(KIZ03055), from Leishan county, Guizhou; 6400030(KIZ003047), from Kunming, Yunnan.­­­

***Mustela strigidorsa*** 5 adult female specimens. 84470(KIZ009702), from Dianjin county, Yunnan; 220034(KIZ011803), from Yuanchun county, Yunnan; 75847(KIZ000260), from Dehong county, Yunnan; 76256(KIZ004720), from Dehong county, Yunnan; 830263(KIZ009703), from Lincang county, Yunnan.
