# Supplementary material for: Revalidation and expanded description of Mustela aistoodonnivalis (Mustelidae: Carnivora) based on a multigene phylogeny and morphology
Source: Ecol Evol. 2023 Apr 18;13(4):e9944. doi: 10.1002/ece3.9944 (PMC10111237; doi:10.1002/ece3.9944)
Supplement: Supplementary file 6 — Table S2 [file ECE3-13-e9944-s003.docx]

| **Table A2. Morphometric data of *Mustela* species (For abbreviates see Materials and method, 2.5 Linear analyses)** | | | | | | | | | | | | | | | | | | | | |
| --- | --- | --- | --- | --- | --- | --- | --- | --- | --- | --- | --- | --- | --- | --- | --- | --- | --- | --- | --- | --- |
| Species | No. | Sex | W | HBL | TL | HFL | EL | PL | BL | MPL | LBO | LBPP | ZB | LTB | GWTB | GNB | HB | OLVR | OHVR | LIOC |
| *M. aistoodonnivalis* | csd2000 | ♀ | 39 | 114.5 | 62.5 | 22.5 | 11.5 | 29.79 | 27.42 | 11.53 | 6.29 | 6.51 | 14.83 | 9.01 | 5.6 | 13.73 | 10.5 | 14.53 | 6.84 | 29.76 |
| *M. aistoodonnivalis* | csd2015 | ♀ | 40 | 150 | 70 | 20 | 12 | 30.14 | 28.78 | 11.48 | 6.48 | 8.76 | 16.32 | 9.71 | 6.02 | 16.49 | 11.97 | 15.12 | 7.07 | 30.74 |
| *M. aistoodonnivalis* | csd2339 | ♀ | 44 | 150 | 67 | 25 | 12 | 33.12 | 30.5 | 13.02 | 6.79 | 8.12 | 16.53 | 9.92 | 6.5 | 16.52 | 12.78 | 16.2 | 8.22 | 32.99 |
| *M. aistoodonnivalis* | csd3528 | ♀ | —— | 150 | 60 | 24 | 10 | 34.16 | 31.73 | 13.64 | 6.93 | 7.94 | 17.28 | 10.36 | 6.53 | 15.63 | 12.22 | 16.71 | 8.28 | 34.32 |
| *M. aistoodonnivalis* | csd3529 | ♀ | —— | 135 | 58 | 21 | 10 | 30.49 | 28.32 | 11.86 | 6.32 | 7.31 | 15.65 | 9.18 | 6.05 | 14.53 | 10.91 | 15.17 | 7.14 | 30.72 |
| *M. aistoodonnivalis* | HS-Ⅱ-013 | ♀ | —— | 124 | 60 | 22 | 12 | 30.45 | 28.46 | 12.09 | 6.38 | 8.01 | 16.08 | 10.33 | 6.34 | 14.4 | 10.71 | 15.37 | 7.28 | 30.66 |
| *M. altaica* | csd891 | ♀ | 89 | 183 | 100 | 31 | 16 | 40.37 | 36.97 | 17.02 | 7.78 | 7.9 | —— | 12.31 | 7.13 | 17.78 | 13.38 | 20.19 | 8.87 | 40 |
| *M. altaica* | csd2003 | ♀ | 130 | 210 | 110 | 33 | 15 | 43.41 | 39.85 | 18.81 | 9.06 | 9.94 | 22.32 | 12.65 | 7.98 | 21.27 | 16.99 | 23.15 | 10.88 | 42.97 |
| *M. altaica* | csd3531 | ♀ | 73 | 190 | 92 | 27 | 11 | 36.54 | 33.72 | 14.76 | 6.67 | 8.96 | 16.49 | 11.03 | 6.34 | 17.85 | 15.68 | 17.6 | 8.45 | 36.19 |
| *M. altaica* | csd1656 | ♀ | 143 | 215 | 92 | 31 | 16 | 40.8 | 38.59 | 17.14 | 7.29 | 8.9 | 18.77 | 12.68 | 7.17 | 19.31 | 16.89 | 20.69 | 8.75 | 41.36 |
| *M. erminea* | yx2051 | ♀ | —— | —— | —— | —— | —— | 56.08 | 54.84 | 26.68 | 14.22 | 13.65 | 31.99 | 15.7 | 10.99 | 28.27 | 24.55 | 37.08 | 16.87 | 59.2 |
| *M. erminea* | 903130568 | ♀ | —— | —— | —— | —— | —— | 45.91 | 44.35 | 20.22 | 10.92 | 10.23 | 26.32 | 14.81 | 9.11 | 21.85 | 24.63 | 25.02 | 11.64 | 48.49 |
| *M. erminea* | 0903049 | ♀ | —— | —— | —— | —— | —— | 60.78 | 60.03 | 31.13 | 16.28 | 16.16 | 37.17 | 17.66 | 13.13 | 33.09 | 25.44 | 40.81· | 19.37 | 63.95 |
| *M. erminea* | 0903yan198 | ♀ | —— | —— | —— | —— | —— | 47.11 | 46.84 | 21.92 | 11.82 | 11.61 | 28.51 | 15.82 | 10.93 | 30.97 | 24.61 | 26.52 | 12.21 | 50.52 |
| *M. erminea* | IOZ26045 | ♀ | —— | —— | —— | —— | —— | 54.66 | 51.77 | 22.66 | 11.67 | 11.55 | 29.33 | 16.88 | 8.89 | 23.33 | —— | 29.42 | 13.11 | 55.33 |
| *M. eversmanii* | SAF20121 | ♀ | —— | —— | —— | —— | —— | 71.81 | 68.55 | 35.72 | 18.19 | 13.61 | 42.34 | 17.81 | 12.85 | 31.12 | 28.95 | 44.42 | 22.12 | 71.64 |
| *M. eversmanii* | SAF20122 | ♀ | —— | —— | —— | —— | —— | 67.69 | 62.84 | 34.44 | 18.37 | 15.88 | 45.49 | 14.89 | 11.41 | 31.65 | 29.47 | 44.51 | 20.21 | —— |
| *M. eversmanii* | SCDX62 | ♀ | —— | 360 | 156 | 50 | 25 | 61.44 | —— | 31.35 | 16.47 | 14.42 | 37.44 | 17.1 | 11.33 | 27.92 | 25.32 | 38.33 | 18.05 | —— |
| *M. eversmanii* | mucuogan02 | ♀ | —— | —— | —— | —— | —— | 71.79 | 67.95 | 35.55 | 18.31 | 12.7 | 42.33 | 18.43 | 15.93 | 32.58 | 24.62 | 44.39 | 21.95 | 72.27 |
| *M. eversmanii* | K004740 | ♀ | —— | —— | —— | —— | —— | 72.23 | 67.75 | 37.13 | 23.42 | 15.66 | 33.32 | 21.14 | 17.58 | 35.17 | 30.65 | —— | —— | 72.56 |
| *M. eversmanii* | K004741 | ♀ | 800 | 360 | 140 | 54 | 25 | 66.18 | 64.29 | 34.79 | 18.2 | 13.7 | 41.01 | 17.43 | 13.87 | 29.54 | 24.63 | 41.2 | 20.2 | 67.73 |
| *M. eversmanii* | XJ001 | ♀ | 663 | 340 | 140 | 52 | 20 | 63.21 | 61.58 | 31.63 | 17.47 | 13.97 | 40.29 | 17.75 | 12.74 | 28.86 | 26.81 | 45.56 | 20.11 | 65.61 |
| *M. kathiah* | SCDX5901 | ♀ | —— | 245 | 160 | 38 | 12 | 49.79 | 47.12 | 21.26 | 10.75 | 13.34 | 26.56 | 14.42 | 7.38 | 9.83 | 5.18 | 13.49 | 14.07 | 50.31 |
| *M. kathiah* | K034552 | ♀ | 176 | 233 | 165 | 40 | 15 | 46.4 | 44.12 | 19.84 | 10.2 | 11.44 | 25.34 | 14.81 | 6.58 | 20.99 | 16.88 | 26.28 | 12.79 | 47.62 |
| *M. kathiah* | K034553 | ♀ | —— | —— | —— | —— | —— | 48.36 | 45.36 | 21.33 | 10.5 | 10.83 | 24.65 | 14.25 | 6.82 | 20.47 | 16.38 | 24.7 | 12.01 | 48.55 |
| *M. kathiah* | K016368 | ♀ | 204 | 267 | 167 | 41 | 16 | 49.95 | 46.23 | 22.22 | 9.75 | 9.43 | 26.14 | 14.43 | 6.54 | 20.78 | 17.01 | 27.88 | 13.49 | 49.46 |
| *M. kathiah* | K003806 | ♀ | —— | 280 | 180 | 45 | 12 | 49.57 | 46.28 | 21.47 | 7.84 | 9.4 | 23.45 | 15.15 | 7.71 | 20.91 | 16.81 | 27.19 | 12.95 | 49.65 |
| *M. kathiah* | K003807 | ♀ | 250 | 260 | 185 | 46 | 21 | —— | —— | 21.52 | 10.01 | 11.82 | 24.41 | —— | —— | 23.86 | 15.64 | 27.06 | 13.72 | 48.45 |
| *M. nivalis* | csd2011 | ♀ | 65 | 170 | 55 | 27 | 11 | 35.17 | 32.73 | 13.87 | 7.69 | 7.03 | 18.07 | 12.53 | 6.86 | 16.02 | 13.05 | 17.86 | 8.35 | 35.45 |
| *M. nivalis* | csd1480 | ♀ | 54 | 183.6 | 54.3 | 25.56 | 9 | 33.47 | 31.26 | 13.73 | 6.76 | 7.46 | 16.77 | 10.53 | 6.5 | 15.13 | 11.93 | 16.67 | 8.01 | 33.49 |
| *M. nivalis* | csd3526 | ♂ | 59 | 158 | 46 | 22 | 12 | 32.12 | 30.18 | 13.46 | 7.69 | 8.28 | 16.37 | 10.66 | 6.1 | 15.13 | 12.57 | 15.88 | 7.45 | 32.3 |
| *M. nivalis* | csd3530 | ♂ | 59 | 157 | 23 | 22 | 11 | 32.25 | 30.27 | 13.56 | 6.85 | 7.11 | —— | 10.9 | 6.35 | —— | 12.77 | 15.82 | 7.76 | 32.56 |
| *M. nivalis* | 13325 | ♀ | 120 | 135 | 35 | 23 | 12 | 30.2 | 27.85 | 11.58 | 5.34 | 7.01 | 15.96 | 10.12 | 5.33 | 15.34 | 12.51 | 14.72 | 7.39 | 30.31 |
| *M. sibirica* | csd3733 | ♀ | 261 | 260 | 147 | 52 | 20 | 51.52 | 47.49 | 22.17 | 9.77 | 12.67 | —— | 16.18 | 10.73 | 23.91 | 22 | 28.15 | 13.48 | 51.25 |
| *M. sibirica* | csd2005 | ♀ | 343 | 270 | 185 | 55 | 25 | 57.66 | 53.63 | 25.4 | 11.76 | 12.95 | —— | 17.52 | 10.57 | 25.13 | 22.79 | 32.42 | 15.38 | 57.92 |
| *M. sibirica* | csd2006 | ♀ | 196 | 240 | 180 | 75 | 20 | 52.15 | 48.14 | 22.93 | 10.76 | 13.06 | 25.43 | 14.76 | 9.09 | 23.11 | 18.67 | 27.94 | 13.11 | 52.02 |
| *M. sibirica* | csd2007 | ♀ | 318 | 280 | 170 | 60 | 20 | 59.2 | 54.77 | 26.65 | 12 | 12.96 | 30.5 | 16.58 | 10.43 | 25.73 | 22.72 | 33.16 | 16.44 | 59.31 |
| *M. sibirica* | csd2008 | ♀ | 279 | 240 | 185 | 46 | 20 | 51.65 | 47.73 | 22.25 | 10.16 | 12.2 | 25.23 | 15.02 | 8.92 | 22.98 | 19.06 | 27.39 | 13.24 | 51.39 |
| *M. sibirica* | csd2379 | ♀ | 425 | 300 | 265 | 55 | 25 | 59.29 | 54.42 | 26.45 | 12.12 | 13.38 | 29.93 | 15.65 | 10.06 | 25.29 | 22.3 | 33.19 | 16.57 | 58.66 |
| *M. sibirica* | HY1 | ♀ | —— | 270 | 185 | 55 | 25 | 59.83 | 55.86 | 27.37 | 11.93 | 11.35 | 32.66 | 17.01 | 10.98 | 24.43 | 21.63 | 33.73 | 16.15 | 59.71 |
| *M. sibirica* | HY2 | ♀ | —— | 240 | 180 | 75 | 20 | 64.41 | —— | 29.45 | 12.89 | 11.13 | 34.58 | 18.11 | 11.39 | 25.87 | 23.59 | 36.57 | 18.27 | —— |
| *M. sibirica* | HY3 | ♀ | —— | 280 | 170 | 60 | 20 | 64.75 | 61.31 | 30.47 | 13.45 | 11.71 | 36.03 | 19.62 | 11.67 | 26.06 | 23.4 | 37.14 | 19.15 | 64.99 |
| *M. sibirica* | CJ-30 | ♀ | —— | —— | —— | —— | —— | 66.68 | 62.89 | 30.29 | 13.85 | 11.59 | 37.01 | 18.75 | 12.5 | 26.69 | 25.5 | 37.78 | 20.01 | 66.68 |
| *M. sibirica* | CJ | ♀ | —— | —— | —— | —— | —— | 66.3 | 62.61 | 29.1 | 14.09 | 12.32 | 37.8 | 18.24 | 13.1 | 27.53 | 25.57 | 38.41 | 20.02 | 67.4 |
| *M. sibirica* | SCDX001 | ♀ | 350 | 301 | 195 | 45 | 15 | 66.68 | 62.24 | 30.87 | 13.66 | 13.01 | 35.13 | 19.13 | 10.25 | 26.73 | 25.48 | 36.85 | 20.63 | 66.67 |
| *M. sibirica* | SCDX202201 | ♀ | —— | 420 | 180 | 31 | 22 | —— | 48.09 | 21.98 | 10.43 | 12.71 | 27.01 | 15.46 | 8.6 | 26.29 | 21.91 | 27.92 | 12.96 | 51.52 |
| *M. sibirica* | 6400030 | ♀ | 382 | 310 | 160 | 50 | 16 | 54.12 | 51.34 | 24.14 | 10.64 | 10.28 | 27.94 | 16.82 | 8.1 | 22.03 | 21.24 | 29.89 | 14.95 | 55.35 |
| *M. sibirica* | 820133 | ♀ | 250 | 262 | 135 | 46 | 18 | 50.1 | 47.72 | 23.4 | 9.71 | 8.42 | 24.39 | 15.63 | 8.18 | 21.05 | 19.66 | 27.34 | 13.55 | 50.99 |
| *M. sibirica* | 640201 | ♀ | 294 | 230 | 156 | 47 | 17 | 53.88 | 51.64 | 24.23 | 9.93 | 9.73 | 26.19 | 16.95 | 8.01 | 21.69 | 20.32 | 29.63 | 14.15 | 54.83 |
| *M. sibirica* | 631350 | ♀ | 350 | 295 | 164 | 47 | 22 | 53.12 | 51.39 | 24.04 | 10.27 | 9.97 | 25.57 | 16.18 | 7.84 | 22,08 | 21.33 | 29.5 | 13.09 | 55.02 |
| *M. sibirica* | 631318 | ♀ | 300 | 275 | 160 | 50 | 18 | 50.17 | 49.76 | 23.99 | 10.54 | 11.38 | 25.67 | 16.65 | 8.08 | 21.74 | 20.54 | 28.58 | 14.13 | 52.67 |
| *M. sibirica* | 88303 | ♀ | 300 | 270 | 148 | 47 | 18 | 51.86 | 49.64 | 23.91 | 10.01 | 11.19 | 26.54 | 16.88 | 7.47 | 22.83 | 19.86 | 28.59 | 13.77 | 53.05 |
| *M. sibirica* | 820199 | ♀ | 250 | 260 | 130 | 44 | 16 | 48.03 | 45.76 | 22.59 | 9.83 | 10.16 | 25.64 | 15.66 | 7.19 | 20.93 | 18.99 | 26.77 | 12.79 | 49.16 |
| *M. sibirica* | 820432 | ♀ | 260 | 280 | 180 | 50 | 22 | 51.98 | —— | 22.39 | 10.04 | 11.72 | 23.08 | 14.98 | 8.41 | 23.95 | 20.21 | 27.82 | 13.79 | —— |
| *M. strigidorsa* | 84470 | ♀ | 300 | 270 | 143 | 41 | 11 | 58.45 | 52.69 | 27.48 | 13.16 | 15.63 | 34.11 | 15.14 | 10.48 | 28.48 | 21.92 | 37.28 | 18.6 | 55.01 |
| *M. strigidorsa* | 220034 | ♀ | —— | —— | —— | —— | —— | 52.09 | 52.15 | 24.11 | 11.91 | 12.88 | 29.83 | 14.88 | 8.71 | 23.96 | 18.02 | 31.63 | 15.28 | 52.18 |
| *M. strigidorsa* | 75847 | ♀ | 343 | 340 | 200 | 54 | 23 | 60.91 | 58.22 | 29.61 | 15.71 | 15.89 | 36.83 | 15.54 | 10.29 | 26.87 | 22.59 | 38.06 | 18.59 | 62.31 |
| *M. strigidorsa* | 76256 | ♀ | 360 | 325 | 185 | 56 | 14 | 60.47 | 57.24 | 28.45 | 14.54 | 15.23 | 33.22 | 17.13 | 10.3 | 26.88 | 20.77 | 35.79 | 18.46 | 61.22 |
| *M. strigidorsa* | 830263 | ♀ | —— | —— | —— | —— | —— | 53.7 | 51.95 | 25.59 | 13.02 | 13.32 | 29.71 | 16.6 | 10.18 | 26.48 | 18.43 | 32.47 | 16.14 | 55.32 |
